# Data-Driven Critical Evaluation of the General Solubility Equation

**DOI:** 10.1021/acs.jcim.6c01182

**Published:** 2026-08-24

**Authors:** Esteban Bertsch-Aguilar, Nahomy Quirós, Frederick Schosinsky, Silvana Pinheiro, José L. Medina-Franco, William J. Zamora

**Affiliations:** † CBio^3^ Laboratory, School of Chemistry, 27915 Universidad de Costa Rica, San Pedro, San José 11501, Costa Rica; ‡ Laboratory of Computational Toxicology and Artificial Intelligence (LaToxCIA), Biological Testing Laboratory (LEBi), Universidad de Costa Rica, San Pedro, San José 11501, Costa Rica; § DIFACQUIM Research Group, Department of Pharmacy, School of Chemistry, 7180 Universidad Nacional Autónoma de México, Mexico City 04510, Mexico; ∥ National Advanced Computing Collaboratory (CNCA), National High Technology Center (CeNAT), Pavas, San José 11501, Costa Rica

## Abstract

Solubility is a widely measured physicochemical property and a highly sought-after descriptor in the evaluation of molecules for drug design and development, medicinal chemistry, and agrochemical sciences. Consequently, a wide range of *in silico* models based on deductive and inductive reasoning have been developed. However, the General Solubility Equation (GSE) stands out as a simple, thermodynamically derived equation to predict intrinsic solubility (log *S*_0_, in mol/L) of molecules from their melting point and *n*-octanol/water partition coefficient (log *P*_N_). Despite its widespread use, the applicability of this equation has not been rigorously evaluated to establish when it can or cannot produce accurate predictions. Therefore, a large-scale evaluation of the GSE was conducted using an extensive database of fully experimental melting point and log *P*_N_ data for 2740, mostly drug-like compounds. A systematic analysis based on cheminformatics descriptors was conducted to identify the primary factors that allow the GSE to achieve predictions within the experimental uncertainty range (±1 log *S* unit). This information has been used to create GSESolver: a supervised machine learning classification model that, based on the selected descriptors, can predict whether the GSE will perform accurately solely from its SMILES string. GSESolver tool can be used by anyone in the scientific community to quickly assess molecule solubility and it was developed under the FAIR principles (Findable, Accessible, Interoperable, and Reusable) in a Google Colab notebook to ensure ease of use. Finally, the utility of the GSESolver tool was exploited in medicinal chemistry applications for the *in silico* prediction of the Maximum Absorbable Dose (MAD) of potential drugs, and in a rational agrochemical design application where solubility is a key factor.

## Graphical abstract

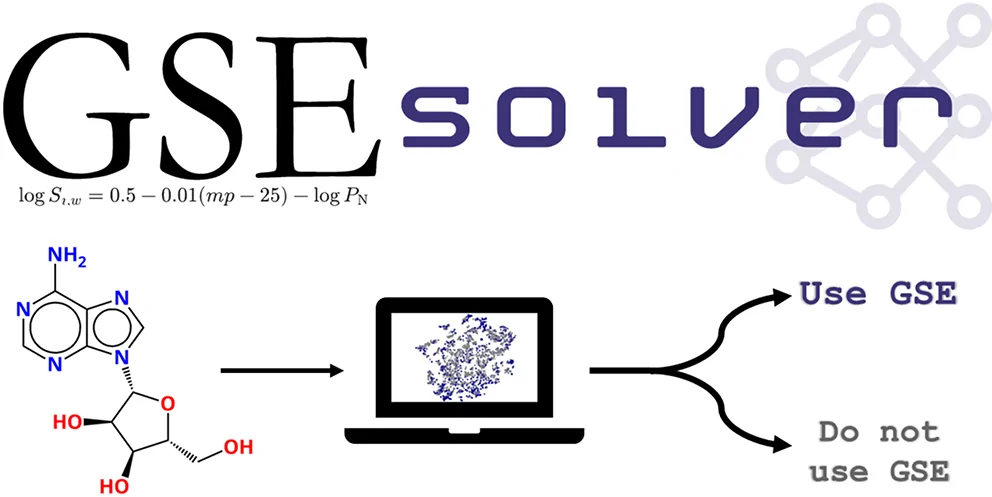

## 1 Introduction

Solubility refers to the composition of a saturated solution in terms of the proportion of a solute in a designated solvent.^1^ From a thermodynamic perspective, it is defined as the maximum concentration at which a substance is at equilibrium between its most stable crystalline form and its dissolved phase under standard conditions. This thermodynamic approach is reflected in the measurement of aqueous solubilities, where pure water is used as the solvent.^2^ In these cases, the pH is not strictly controlled, as the solute may undergo protonation or deprotonation processes until equilibrium is reached at a specific pH. In this context, pH control has posed a challenge for the application of aqueous solubility in drug design and development. Consequently, there is growing interest in the measurement of apparent solubility, which involves determining the solubility of a substance at a controlled pH by using buffers.^2^ Alternatively, in the early phases of drug discovery, kinetic solubility is often measured. This is defined as the maximum concentration at which a substance begins to precipitate when a 10 mM solution of the solute in dimethyl sulfoxide (DMSO) is added to a phosphate buffer at pH 7.4.^3^ Nevertheless, this technique faces challenges due to the formation of equilibria with amorphous phases of the solute and cases of supersaturation caused by acid–base interactions of the solute.^4^

Apparent solubility enables the determination of complete solubility profiles for substances, where the value varies according to the acid–base behavior of the solute, as described by the Henderson–Hasselbalch equation.^2,5^ In such cases, determining the apparent solubility of substances by adjusting the pH so that the solute exists in its neutral species has become highly relevant. This has been termed intrinsic solubility (*S*_0_).^6^ This means that *S*_0_ refers specifically to the solubility of the neutral, un-ionized form of a compound in water. For ionizable molecules (acids, bases, and amphoteric species), *S*_0_ exists at a fixed pH value, while the measured apparent solubility varies as a function of pH. *S*_0_ determination has achieved crucial importance for drug candidates, since poor aqueous solubility can lead to poor absorption and thus inefficient performance.^7^ Therefore, estimating a compound’s lowest possible solubility value provides invaluable information about its potential bioavailability.

The measurement of intrinsic solubility in small molecules has gained significant importance and application across various scientific fields, especially *in vitro* and *in vivo* studies during the early stages of drug discovery.^8^ Drug solubility is directly linked to its bioavailability, with the general understanding that highly soluble substances with high permeability are more likely to have fewer biocompatibility issues.^6,9^ This parameter estimates the dissolution capacity of a drug and assists in determining the maximum absorbable dose (MAD) in the intestine.^6,10^ Similarly, in drug development, knowledge of solubility is essential for dose selection, experimental design to identify salt/crystalline forms, development of analytical methods, and creation of manufacturing protocols.^8,11^ Furthermore, solubility is a relevant parameter in the rational design of modern agrochemicals, with industries conducting studies to determine the experimental solubility of insecticides, fungicides, and herbicides.^12,13^

Experimental techniques such as the Shake-Flask or CheqSol method have been widely used for the determination of apparent (and intrinsic) solubility of solutes.^14^ However, the increasing demand for quick screening values has promoted the significant development of *in silico* solubility prediction models. Deductive computational models employ theoretical calculations to determine the Gibbs free energy of solubility (Δ*G*_sol_), typically by resolving a thermodynamic cycle in which the crystalline particles of the solute undergo a sublimation process (Δ*G*_sub_), followed by solvation in the solvent (Δ*G*_solv_).^15^ Molecular dynamics (MD) simulations have been reported for this purpose using various force fields such as COMPASS, AMBER, GROMACS, and OPLS4.^16–19^ Similarly, quantum mechanical (QM) models have utilized polarizable continuum models to simulate the solvation process with reliable results.^19–23^ However, theory-based models oftentimes require an elevated computational cost. Additionally, the quantification of molar solubility (or otherwise log* S*) is not always reported.^16,23^ In general, deductive methods have not been as widely employed in solubility prediction. In contrast, data-driven inductive methods have been far more extensively studied. Quantitative structure–property relationship (QSPR) models, part of a broader general concept Structure–Property Associations—SPA^24^ provided the first predictive models of, through the use of databases and chemoinformatic descriptors at the one-dimensional (1D, atomic), two-dimensional (2D, molecular), and three-dimensional (3D, surface) levels.^9,25–33^ Furthermore, the creation of curated solubility databases, such as OCHEM,^34^ AqSolDB,^35^ or BigSolDB,^36^ in conjunction with the development of chemoinformatics libraries like RDKit,^37^ RCDK,^38^ and OpenBabel^39^ and the increasing accessibility of packages in the public domain such as Scikit Learn^40^ and TensorFlow,^41^ have fostered the broad development of machine learning (ML) predictive models.^42–48^

In addition to the data-intensive nature of ML approaches, the intrinsic solubility of substances can also be estimated using a simpler, thermodynamically derived equation: the General Solubility Equation (GSE), proposed by Jain and Yalkowsky.^49^ The GSE connects the solubility equilibrium of organic molecules through a thermodynamic cycle, relating a compound’s solubility to its fusion energy via a theoretical “supercooled liquid” state. In addition, the authors suggest using an equilibrium in an *n*-octanol/water system to simplify this relationship, since it is assumed that small organic molecules are generally miscible in *n*-octanol. As a result, the GSE indicates that log* S*_0_ can be calculated through its melting point (mp), and its partition coefficient in an *n*-octanol/water system (log* P*_*N*_):^49^

1 $$\log S_{0}=0.5-0.01\left(\mathrm{mp}-25\right)-\log P_{N}$$

Equation 1 emphasizes that the GSE estimates the intrinsic solubility of molecules. Consequently, the GSE, which is derived under the assumption of a neutral solute, is strictly applicable to nonelectrolytes or to ionizable compounds evaluated at a pH where the neutral form predominates. This limitation should be considered when applying GSE-derived log *S*_0_ calculations to nonneutral conditions, where the apparent solubility can be then estimated using the Henderson–Hasselbalch equation.^50^

Jain and Yalkowsky analyzed a set of 580 organic molecules with known experimental mp values and used calculated parameters to assess the performance of the GSE. They compared the average absolute estimation error to that of a previously derived equation by Yalkowsky and Valvani,^51^ finding that the new model improved accuracy by about 46% over the earlier version.^49^

The predictive performance of the GSE can be benchmarked against a broad landscape of computational solubility models in the literature, encompassing QM, MD, QSAR/QSPR, and ML approaches, where GSE calculations have achieved root-mean-square error (RMSE) values of 0.25–0.80 log units.^49,51–53^ QM-based methods such as COSMO-RS have reported RMSE values in the range of 0.66–1.55 log units depending on the chemical space evaluated, while classical MD simulations have generally been limited to solubility classification rather than quantitative prediction, or have achieved RMSE values exceeding 1.0 log unit for nonaqueous systems.^16–23^ Moreover, these computational methods based on theory oftentimes require high computational costs. QSAR/QSPR models typically yield RMSE values between 0.5 and 1.4 log units within their respective training domains,^26–33^ and contemporary ML models—including graph neural networks and attention-based architectures such as SolTranNet—have reported RMSE values as low as 0.36–0.95 log units on curated data sets.^42–48,54–57^ While the best-performing ML models achieve lower errors than the GSE on their respective benchmarks, it is critical to note that these comparisons are not equivalent: each ML model was trained and evaluated within a specific chemical space, and performance degrades substantially when applied outside that domain.^47,54,58^ In contrast, the GSE requires no training data and operates solely on two experimentally or computationally accessible parameters (mp and log *P*_*N*_), making it applicable to any compound for which these values are available, irrespective of structural class.

The aim of this study is 2-fold. First, to construct the largest experimentally derived database to date for evaluating log *S*_0_ using the GSE (SolCbio3 Database). Second, to develop GSESolver, a tool built under the FAIR (Findable, Accessible, Interoperable, and Reusable) principles,^59^ programmed in Google Colab for early-stage drug design that predicts the reliability of GSE-based log *S*_0_ estimates for a given drug-like molecule. The accuracy within the applicability domain of the GSE does not consist in the equation’s principal limitation, but rather the absence of a principled, data-driven criterion to determine *a priori* whether a given molecule belongs to that domain. It is precisely this gap that GSESolver addresses: rather than replacing the GSE, it provides a validated binary classifier that predicts whether the GSE’s thermodynamic assumptions are satisfied for a given molecule, thereby enabling users to apply the GSE with confidence or redirect their analysis to alternative methods when it is likely to fail. Unlike QSPR and ML models, whose accuracy degrades outside the structural space of their training data, the GSE + GSESolver combination imposes no such constraint, where GSESolver predicts the applicability from SMILES alone, and the GSE itself requires no training data set.

## 2 Methodology

### 2.1 Data Collection, Classification, and GSE Evaluation

#### 2.1.1 SolCbio3Database

This study involved the construction of the SolCbio3Database: an open database that compiles experimentally determined intrinsic solubility (log* S*_0_, in mol/L), melting point (mp, in °C), and neutral octanol–water partition coefficient (log* P*_*N*_) values for small organic molecules, as reported in peer-reviewed literature. The data collection prioritized primary literature sources that specifically evaluated molecules using the GSE. These sources often also reported mp and log* P*_*N*_ values. For studies reporting apparent solubility as a function of pH, only the lowest solubility value for each molecule was collected, corresponding to the intrinsic solubility (i.e., the value at the lowest pH for acids and the highest pH for bases).^5,52,60–63^ We acknowledge, however, that for amphoteric molecules the minimum apparent solubility may not coincide with the point of maximum neutralization, but rather with the isoelectric point at which zwitterionic species are most abundant. Nevertheless, this experimental minimum roughly corresponds to the theoretical log*S*_0_, based on matching experimental data to the Henderson–Hasselbalch-derived equation for apparent solubility.^61^ Sulfadimethoxine (SolCbio3-385) and Cefadroxil (SolCbio3-899) were the only amphoteric compounds added under this criterion.

This primary literature data was supplemented with entries from publicly available solubility databases:

1. Wiki-pS0 Database: This database comprises 7542 log *S*_0_ measurements for 3667 molecules, integrating a thorough literature compilation, external databases, FDA-approved drug data (2016–2022), and in-house measurements.^64^ For each molecule, the name, SMILES strings,^65^ log* S*_0_, and mp were extracted. To ensure homogeneity, only the measurement closest to 298 K was retained for each compound.
2. AqSolDB: This source provided aqueous solubility data (in mol/L, at pH ≈ 7 and *T* ≈ 300 K) for 9982 molecules.^35^
3. BigSolDB2.0: This database contains 103,944 solubility values for solutes in diverse solvents at various temperatures.^36^ For our purposes, only aqueous solubility values closest to those at 298 K were extracted.

For AqSolDB and BigSolDB2.0, a filter was applied using the piChemist package in Python^66^ (see Data Availability for scripts) to remove molecules with ionizable groups, under the assumption that the reported aqueous solubility for a nonionizable molecule approximates its intrinsic solubility. The SMILES codes and log* S*_0_ were collected for the remaining molecules from these databases. The MolVS 0.1.1 package was used to standardize the SMILES codes, to detect repeated molecules among SolCbio3 Database.^67^ When multiple experimental log *S*_0_ values were available for the same compound, a quality-based selection procedure was applied. For sources reporting measurements at variable temperatures, the value recorded closest to 298 K (25 °C) was retained, consistent with the thermodynamic conditions assumed by the GSE. For compounds with measurements from multiple sources at equivalent temperatures, the value from the source with the highest experimental rigor was prioritized in the following order: (1) values from our primary literature compilation, (2) Wiki-pS0 database, (3) AqSolDB, and (4) BigSolDB2.0. All compounds with overlapping entries across two or more source databases were identified by standardized SMILES matching. A total of 172 compounds presented log* S*_0_ measurements from multiple sources, for which the standard deviation (SD) across available values was computed as an empirical estimate of interlaboratory measurement variability (Figure S1). Most overlapping compounds exhibited SDs below 0.5 log units, consistent with interlaboratory reproducibility reported in the literature for intrinsic solubility measurements.^68–70^ 14 compounds displayed SDs exceeding this range and were resolved by retaining the log* S*_0_ value from the primary literature compilation, following the hierarchical source-priority criterion. The complete per-compound SD data are provided as a CSV file (overlapped_logS.csv), along with workflow documentation and scripts in the GitHub repository (https://github.com/cbio3lab/GSESolver). Furthermore, an in-house database, with 14,040 experimental log *P*_*N*_ values, was used to obtain the lipophilicity for 2401 molecules from our database.^71^ Finally, remaining mp and log *P*_*N*_ values were collected from PubChem.^72^ It is well documented that PubChem’s log *P*_*N*_ entries are heterogeneous in provenance, and that calculated values—including those computed by XLogP3 and related algorithms—may appear within the experimental properties section without consistent labeling. To ensure that only experimentally determined log* P*_*N*_ values were incorporated into the SolCbio3 Database, each PubChem-sourced entry was individually inspected for source attribution. Entries explicitly labeled as predicted or computed, as well as entries carrying no identifiable source label and therefore of unverifiable experimental provenance, were discarded from the data set. Only log* P*_*N*_ values with a traceable experimental source were retained. This curation step ensured that all log *P*_*N*_ values in the SolCbio3 Database, regardless of their primary source, reflect experimentally measured partition coefficients.

After data curation, the SolCbio3Database contains experimental log *S*_0_, log *P*_*N*_, and mp data for 2740 organic molecules. This data set is openly available in Zenodo (https://zenodo.org/records/18624099).^73^

The data from the SolCbio3 Database was used to calculate log* S*_0_ values for every molecule using the GSE (eq 1). The accuracy of each calculation was evaluated with the Δlog *S* parameter (eq 2):

2 $$\Delta \log S=\left|\log S_{\exp}-\log S_{\mathrm{GSE}}\right|$$

Furthermore, experimental and calculated log* S*_0_ values were correlated to evaluate the general accuracy of the GSE, in which Pearson’s correlation (eq 3), root-mean-square error (eq 4), mean absolute error (eq 5), and mean signed error (eq 6) were calculated.

3 $$r^{2}={\left[\frac{\sum_{i=1}^{n}\left(x_{i}-\mathrm{x̅}\right)\left(y_{i}-\mathrm{y̅}\right)}{\sqrt{\sum_{i=1}^{n}{\left(x_{i}-\mathrm{x̅}\right)}^{2}}\sqrt{\sum_{i=1}^{n}{\left(y_{i}-\mathrm{y̅}\right)}^{2}}}\right]}^{2}$$

4 $$\mathrm{RMSE}=\sqrt{\frac{1}{n}\sum_{i=1}^{n}\left(y_{i}-\hat{y_{i}}\right)}$$

5 $$\mathrm{MAE}=\frac{1}{n}\sum_{i=1}^{n}\left|y_{i}-\hat{y_{i}}\right|$$

6 $$\mathrm{MSE}=\frac{1}{n}\sum_{i=1}^{n}y_{i}-\hat{y_{i}}$$

#### 2.1.2 External Data Sets

Two external databases were constructed as practical applications of the GSE classification ML model. Initially, a limited data set of drugs with experimentally reported parameters for the calculation of their MAD was compiled (eq 7).^10,74,75^

7 $$\mathrm{MAD}=S\times k_{a}\times \mathrm{WV}\times \mathrm{TT}$$

where *S* represents its solubility (in mg/mL), *k*_a_ its absorption rate constant (in min^–1^), WV its water volume in the organ of interest (mostly the small intestine or the mouth, in mL), and TT its transit time (in min). Data from a sample of FDA-approved drugs with publicly available information for MAD determination—including Azithromycin, Rizatriptan, Vardenafil, Propranolol, Verapamil, and Nicotine—were collected. In addition, mp and log *P*_*N*_ values were acquired from PubChem. Considering that the solubility values are reported at a pH of 6.5, the web tool LiProS was used to determine the most appropriate equation to calculate its pH-dependent distribution coefficient (log* D*_6.5_) from its log *P*_*N*_, where their required p*K*_a_ values were obtained from ChemAxon’s p*K*_a_ plugin.^76,77^ This data was used to calculate the log* S*_0_ using eq 1 at the adjusted pH value and determine an *in silico* MAD value, which was compared to its experimental MAD.^78^

A small agrochemical GSE database was also constructed. Zhang et al. provides a list of experimental water solubility (in ppm), mp (in °C), and log* P*_*N*_ values for 87 agrochemical compounds (chewing pest insecticides (CPIS), sap-feeding pest insecticides (SFPI), broad-spectrum fungicides (FUNG), and systemic postemergency herbicides (PEHS)).^12^ These values were collected and used to calculate their GSE values (eq 1).

### 2.2 Descriptor and Binary Response Determination for Machine Learning Classification Modeling

A concrete Δlog* S* value can be set as a threshold to determine if the GSE achieved an acceptable prediction. This delimiter was set with the following criteria:

1. This threshold should align with the experimental uncertainty range (±1 log *S* unit).
2. This threshold should provide a balanced data set, where a significant number of molecules are classified within both the positive group (where the GSE provides a good fit) and the negative group (where the GSE provides poor fitting).
3. The threshold should separate the groups in such a way that a considerable number of descriptors exhibit significant differences in their values between both groups.

Point (1) was approached with a thorough literature search of experimental results of intrinsic solubility measurements, with their respective uncertainties, and interlaboratory reproducibility. Point (2) was evaluated through an iterative argument, in which an arbitrary threshold Δlog *S* = *i* was chosen, where *i* ∈ [0; 2] with a 0.1 increment after each iteration. The data is divided according to the criterion of Δlog* S* ≤ *i* (which were labeled “0” for YES and “1” for NO).

Finally, point (3) was addressed by calculating physicochemical and topological descriptors using RDKit,^37^ Openbabel,^39^ RCDK,^38^ Jazzy,^79,80^ and ConjugaR^81^ (an in-house tool). Null, constant, and intercorrelated descriptors (*r* ^2^ > 0.6) were eliminated. Then, the data set is separated through the same iterative algorithm from point (2), and the means for each group are calculated for each descriptor, for which the Δ*x̅* parameter and its standard uncertainty δ_Δ*x̅*_ are calculated (eqs 8 and 9):

8 $$\Delta \mathrm{x̅}=\left|{\mathrm{x̅}}_{\mathrm{YES}}-{\mathrm{x̅}}_{\mathrm{NO}}\right|$$

9 $$\delta_{\Delta \mathrm{x̅}}=\sqrt{{\left(\frac{s_{YES}}{\sqrt{N_{YES}}}\right)}^{2}+{\left(\frac{s_{NO}}{\sqrt{N_{NO}}}\right)}^{2}}$$

where *s* represents the standard deviation of a descriptor and *N* the number of molecules in each group. The descriptors in which δ_Δ*x̅*_ > Δ*x̅* are eliminated due to a lack of an objective criterion for a difference between means for this descriptor. Next, a univariate feature selection (UFS) was performed through a two-tailed Welch’s *t*-test to identify significant mean differences in descriptors between the two groups. This specific test accommodates non-normal distributions and unequal variances.^82^ The descriptors that failed to reject the null hypothesis with a 95% confidence interval were excluded for each iteration of Δlog* S* = *i*.

The 101 descriptors selected (Table S1,^83–87^ Figure S2) via Welch’s *t*-test, along with the selected Δlog* S* = 1 threshold value (see Section 3), were used to analyze SolCbio3 Database’s chemical space across both groups. Visual examination began by plotting distributions for Lipinski’s^9^ and Veber’s^88^ parameters (molecular weight, hydrogen bond donor/acceptor (HBD/HBA) count, clog *P*, Topological Polar Surface Area (TPSA)), plus experimental log* S*_0_ values. Next, the descriptors enabled principal component analysis (PCA) and *t*-distributed stochastic neighbor embedding (*t*-SNE) analyses to qualitatively explore for visual group separation. Then, RDKit’s “BRICS.decompose” algorithm identified each group’s most common molecular fragments through their SMILES codes. Finally, a differential frequency analysis was conducted on the BRICS fragments comparing enrichment between the positive and negative classes. Fragment occurrence counts were tabulated separately for the positive (*n*_pos) and negative (*n*_neg) classes. For each unique fragment observed across the data set, a 2 × 2 contingency table was constructed from its presence/absence counts in each class, and a two-tailed Fisher’s exact test was applied to evaluate whether the fragment’s frequency differed significantly between classes.^89^ Fragments with a combined occurrence below 5 molecules were excluded prior to testing to avoid unreliable *p*-value estimates from sparse contingency tables. Because this procedure involves simultaneous hypothesis testing across all observed fragments, raw *p*-values were corrected for multiple comparisons using the Benjamini–Hochberg false discovery rate (FDR) procedure, and fragments with an adjusted *p*-value (*q*) below 0.05 were considered statistically significant.^90^

### 2.3 Machine Learning Classification Models for Assessing GSE Viability

#### 2.3.1 Molecular Fingerprints Generation and Recursive Feature Elimination

Morgan fingerprints were included as descriptors. Using RDKit’s AllChem module, 1024 molecular representations (radius = 2) were calculated, totaling 1125 descriptors per molecule. Recursive feature elimination (RFE) was then applied with an untuned Random Forest model, removing the 50 least important descriptors at each step. The RFECV module from Scikit Learn^91^ determined descriptor importance via Gini contributions using 3-fold cross-validation. RFE showed maximum performance with 75 selected descriptors, which were used to train our classification ML models (Table S2).

#### 2.3.2 Sampling, Model Training, and Validation

First, a rule-based decision model was established, based on SolCbio3 Database mp and TPSA values between the positive and negative classes. For that, a univariate threshold optimization procedure was performed. For each descriptor independently, every unique observed value was considered as a candidate threshold. At each candidate threshold *t*, samples were assigned to the negative class if the descriptor value exceeded *t* (i.e., *x* > *t*), and to the positive class otherwise. For every resulting binary classification, the confusion matrix was computed, from which the sensitivity (eq 10) and specificity (eq 11) were calculated. The optimal threshold was selected by maximizing Youden’s *J* statistic (eq 12).^92^ This index identifies the value that maximizes the balance between correct positive and correct negative predictions. The procedure was applied independently to mp and TPSA, yielding distinct optimal thresholds for each descriptor.

10 $$\mathrm{sensitivity}=\frac{\mathrm{TP}}{\mathrm{TP}+\mathrm{FN}}$$

11 $$\mathrm{specificity}=\frac{\mathrm{TN}}{\mathrm{FP}+\mathrm{TN}}$$

12 J=sensitivity+specificity−1

A training set (∼80%, or 2192 molecules) and a test set (∼20%, or 548 molecules) were randomly chosen, with a stratification algorithm applied to maintain a balanced representation of each group in both sets. Moreover, a validation set was also sampled with molecules of the training set (∼20% of the training set, or 439 molecules). The training set was utilized to develop several supervised and reinforcement models. Specifically, a Random Forest Classifier (RFC), a Support Vector Machine (SVM), and a Naïve Bayes (NB) model were created using Scikit Learn’s RandomForestClassifier, SVC, and GaussianNB modules, respectively.^40^ Additionally, an eXtreme Gradient Boosting (XGBoost) model was built using the XGBoost Python package.^93^ Hyperparameters for the RFC, SVM, and XGBoost models were optimized with Scikit Learn’s RandomizedSearchCV module. An Artificial Neural Network (ANN) was also trained with the TensorFlow package.^41^ Further details regarding the hyperparameter tuning for the RFC, SVM, and XGBoost models, as well as the mathematical formulation of the Naïve Bayes model and the ANN’s layer architecture, are available in the Supporting Information. Furthermore, an RFC + XGBoost ensemble model was employed through soft voting, wherein the predicted class probabilities from each model were averaged and a new optimal decision threshold was applied.

For every model, the optimal decision threshold was independently determined using the validation set, for which the sensitivity (eq 10) and specificity (eq 11) were calculated and used to maximize Youden’s *J* statistic (eq 12).^92^ This index identifies the cutoff that maximizes the vertical distance between the receiver operating characteristic (ROC) curve and the diagonal line of no discrimination, thereby balancing the trade-off between the true positive rate and the false positive rate. Specifically, all probability thresholds spanning the [0, 1] interval were iteratively evaluated against the validation set. The threshold yielding the highest *J* value was selected for that model.

Each model with an optimized threshold was evaluated using the test set. Their performance was measured using confusion matrices, from which accuracy (eq 13), sensitivity (eq 10), specificity (eq 11), *F*_1_ (eq 14), and normalized Matthews correlation coefficient (nMCC) scores (eq 15) were derived.^94^ To further evaluate model robustness, a 10-fold cross-validation was conducted using the cross_val_score and KFold functions from Scikit Learn’s model_selection module. In addition, the area under the ROC curve was additionally computed for each model, for which their respective area under the curve (AUC) were calculated.

13 $$\mathrm{accuracy}=\frac{\mathrm{TP}+\mathrm{TN}}{\mathrm{TP}+\mathrm{FP}+\mathrm{TN}+\mathrm{FN}}$$

14 $$F_{1}=\frac{2\mathrm{TP}}{2\mathrm{TP}+\mathrm{FP}+\mathrm{FN}}$$

15 $$n\mathrm{MCC}=\left(\frac{\mathrm{TP}\cdot \mathrm{TN}-\mathrm{FP}\cdot \mathrm{FN}}{\sqrt{\left(\mathrm{TP}+\mathrm{FP}\right)\left(\mathrm{TP}+\mathrm{FN}\right)\left(\mathrm{TN}+\mathrm{FP}\right)\left(\mathrm{TN}+\mathrm{FN}\right)}}+1\right)\times \frac{1}{2}$$

#### 2.3.3 SHAP Analysis to Determine Model Interpretability

Shapley Additive exPlanations (SHAP) analysis was conducted to rigorously investigate the influence of individual descriptors on model decision-making. The SHAP package in Python was utilized, wherein the Shapley value (ϕ) is calculated for each descriptor through an iterative methodology (eq 16):^95,96^

16 $$\phi_{i}=\sum_{S\subseteq F\left\{i\right\}}\frac{\left|S\right|!\left(\left|F\right|-\left|S\right|-1\right)!}{\left|F\right|!}\left[f_{S\cup \left\{i\right\}}\left(x_{S\cup \left\{i\right\}}\right)-f_{s}\left(x_{s}\right)\right]$$

where ϕ_*i*_ denotes the average marginal contribution of a given descriptor *i* on the model’s predictions. The RFC model served as the basis for this analysis, in which all the possible combinations of subsets of descriptors *S* ⊆ *F* were trained (where *F* represents the complete set of descriptors). For each selected subset *S*, models *f*_*S*∪{*i*}_ (including descriptor *i*) and *f*_s_ are trained to quantify the effect of *i* on model decisions.^95^ The exact Shapley values were computed using the TreeExplainer algorithm, which exploits the internal structure of decision trees to obtain exact solutions to eq 16 without the sampling approximations required for model-agnostic explainers.^95,96^

To mitigate the risk of SHAP estimates reflecting training set-specific patterns rather than generalizable feature contributions, SHAP values were computed within a 10-fold cross-validation scheme. In each of the 10 folds, a TreeExplainer was instantiated using the RFC fitted on the corresponding training partition, and SHAP values were calculated exclusively for the held-out validation samples of that fold. The resulting per-fold SHAP values were assembled into a single matrix aligned to the original sample indices such that every compound in the SolCbio3 Database received a SHAP value computed from a model that had not seen that compound during training. This procedure yields an out-of-fold SHAP estimate for the entire data set while avoiding the optimistic bias associated with explaining a model on the same data used to fit it.

The most influential descriptors were illustrated using a summary plot of the Shapley values, and the model’s decision pathway was further illustrated using waterfall plots for two representative compounds: one correctly classified as GSE-viable and one correctly classified as GSE-nonviable.

### 2.4 GSESolver: A User-Friendly Google Colab Notebook to Assess the Plausibility of the GSE for Small Molecules

GSESolver (General Solubility Equation Solver) was developed as an open-access Google Colab Notebook for online access to the trained ML model, which provides the following workflow:

1. Tools Installation: These steps require only the execution of designated cells by the user; no additional input is necessary. The process will include the following:
  - Install all necessary Python packages to ensure successful notebook operation.
  - Import relevant scripts and RFCs from the GitHub repository.
  - Assign functions for calculating molecular descriptors and fingerprints to molecules that will be imported into subsequent steps.
2 Molecule Importation: GSESolver offers two options for importing molecules into the notebook:
  - Manual Entry: For small numbers of molecules, users may utilize a manual entry cell. Upon execution, a text box labeled “_Enter the SMILES of your molecules_” will appear. Users should enter the SMILES codes of their molecules of interest, separated by spaces (e.g., “CC­(=O)C CC­(=O)­NC1 =CC=C­(C=C1)­O” for acetone and paracetamol).
  - Excel File Import: For larger data sets, GSESolver allows molecule import via an Excel (.xlsx) file. The file must first be manually uploaded to the Colab environment. After executing the relevant cell, a prompt will request the file’s name. The Excel file should contain a column of SMILES codes, with the header titled “smiles” (capitalization is not required).
3 Output Generation: The RFC processes the imported molecules and predicts their GSE viability. The results are compiled into a .csv file (GSESolver_results.csv), which includes each molecule’s SMILES code and its corresponding model prediction in the “prediction” column. The output will indicate either “Use GSE” or “Do not use GSE” based on the RFC’s analysis. This .csv file is then automatically downloaded to the user’s computer.

## 3 Results and Discussion

### 3.1 Large-Scale Evaluation of the General Solubility Equation

The data collected in the SolCbio3Database was evaluated through the GSE (eq 1). Figure S3 (left) presents the distribution of Δlog* S* values, revealing that the GSE showed absolute errors for most molecules that lie between 0 and 2 log units. Consequently, a rigorous determination of a threshold value that classifies GSE calculations as reliable or not was necessary. Our iterative UFS descriptor selection among a wide range of Δlog *S* thresholds is illustrated in Figure S3 (right).

A delimiter of Δlog *S* = 1 was selected as the classification boundary by three convergent criteria:

- Experimental uncertainty alignment: Interlaboratory comparisons of intrinsic solubility measurements consistently report standard deviations of ±0.5–1 log units.^68–70^ Therefore, a threshold of Δlog* S* = 1 corresponds to the outer bound of experimental measurement uncertainty, defining “accurate” predictions as those falling within the combined uncertainty of the GSE equation and the experimental reference value.
- Data set balance: As illustrated in Figure S3, Δlog *S* = 1 yields a 64:36 positive/negative class ratio, which is workable for all evaluated ML algorithms without synthetic oversampling. More stringent thresholds, such as Δlog* S* = 0.5, might produce an imbalanced data set (∼30% positive class). Otherwise, a more lenient threshold (e.g., Δlog *S* = 1.5) might result in a substantially imbalanced data set for the negative class, with an amount of 83% of molecules classified as accurate predictions. Such imbalance raises concerns in implementations of classification ML models, where the minority class tends to be misclassified with most available methods.^97^
- Descriptor differentiation power: Our iterative UFS analysis (Figure S3, right) confirms that Δlog* S* = 1 yields 101 significantly different descriptors between classes, which lies among the highest discrimination power across the tested range without the extreme class imbalance of Δlog* S* = 1.5. While Δlog* S* = 0.5 is chemically more conservative, it reduces the number of differentiating descriptors and creates a training set with limited positive-class examples, reducing a model’s potential ability to learn the characteristics of molecules in relation to their estimation capabilities with the GSE.

Therefore, this analysis concluded that Δlog *S* = 1 stands as a more reliable differentiator between labels, in which:

- If Δlog* S* ≤ 1, the molecule is assigned to the positive class (indicating an accurate GSE calculation).
- If Δlog* S* > 1, the molecule is assigned to the negative class (indicating an inaccurate GSE calculation).

Figure 1a illustrates the correlation between GSE-calculated and experimentally determined intrinsic solubility values within the SolCbio3Database. The evaluation demonstrates that the GSE exhibits notable accuracy for the positive class (indicated in blue), as evidenced by minimal error metrics. The negative class (displayed in gray) constitutes ∼36% of the data set and includes several entries with significant discrepancies between experimental and calculated values. Consequently, a residual plot was produced for the negative class (Figure 1b). This analysis reveals a relative tendency for the GSE to overestimate intrinsic solubility values, suggesting the presence of systematic error in the underlying equation (eq 1). The derivation of the GSE incorporates several simplifications, such as estimating molar fusion entropy via Walden’s rule and assuming miscibility of an organic supercooled liquid solute in *n*-octanol, both of which may introduce systematic inaccuracies. Empirical corrections to the GSE, such as incorporating or modifying variables like TPSA, have demonstrated improved predictive performance in smaller data sets of small organic molecules.^53^ Nonetheless, the optimization of the GSE is beyond the scope of this study, which instead focuses on identifying molecular characteristics that influence the suitability of the GSE as a predictive tool. To examine whether the systematic positive bias observed in Figure 1b could be attributed to nonlinear coupling between the two GSE input parameters, we performed a regression analysis of Δlog *S* against mp, log* P*_*N*_, and their cross-product (mp × log* P*_*N*_). Although a statistically significant interaction was found (*F* = 17.53, *p* = 2 × 10^–5^), this interaction implies a minimal correlation to the Δlog* S* of the data set, as shown in Figure S4, where no significant tendency in the deviation is seen by mp and log* P*_*N*_. Additionally, the interactions also implied a 0.63% increase in the correlation of the variance of the data (*r*_no interaction_^2^ = 0.0115; *r*_interaction_^2^ = 0.0178), which implies that the deviation from the GSE is most likely derived from a multivariate problem, rather than nonlinear interactions between eq 1 variables.

**Figure 1:**
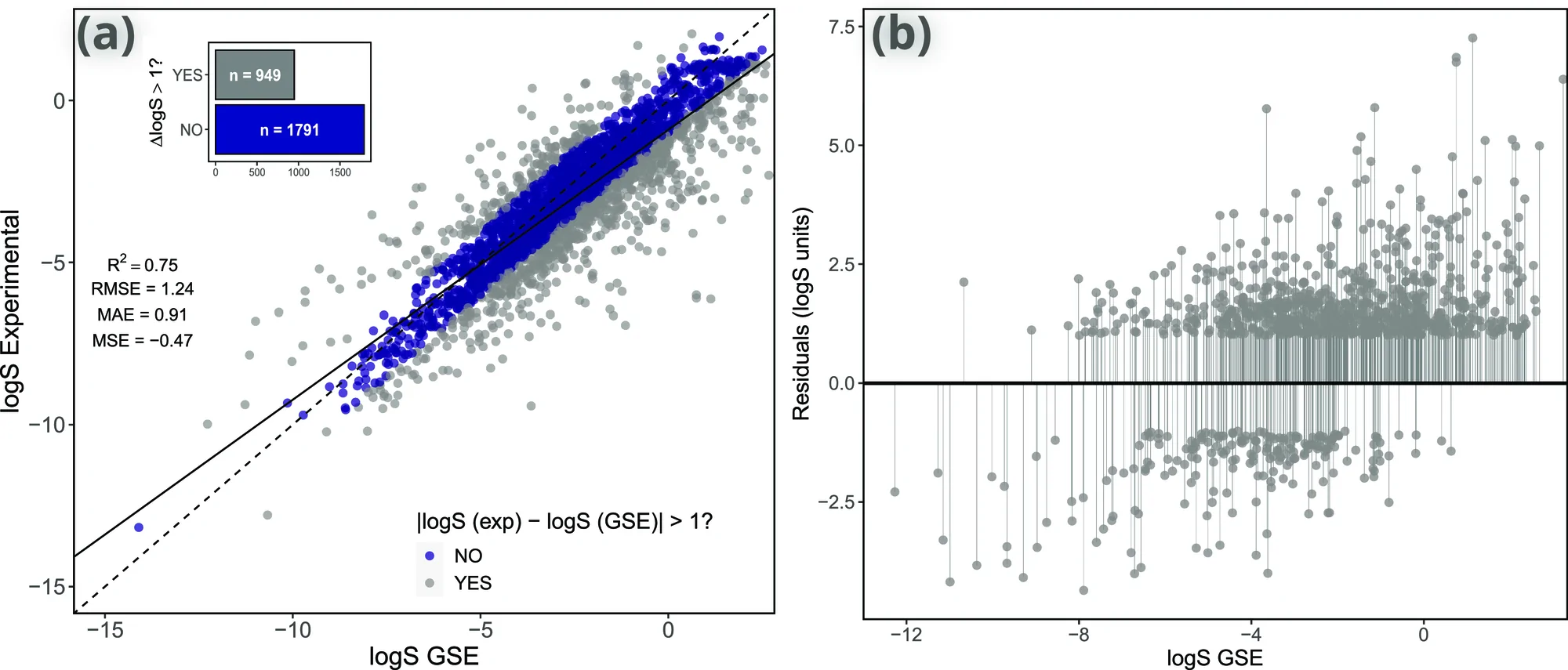
(a) Evaluation of GSE predictions of the SolCbio3 Database as a function of their log* S*_exp_ value. Data points where Δlog *S* ≤ 1 are labeled in blue (positive class), and Δlog *S* > 1 in gray (negative class). The continuous line represents the linear fit of the data, whereas the dotted line represents the identity function (*y* = *x*). A bar plot is included indicating the ratio of accurate and inaccurate GSE predictions. (b) Residual plot of data points assigned to the negative class.

A visual representation of the chemical space of the database was performed as a first approach to differentiate molecular characteristics between both classes. An initial assessment was visualized through the distribution values of Lipinski’s rule of five properties (Figure 2a), where most molecules showed compliance with small drug-like molecules’ characteristics (MW < 500 g/mol, HBA < 10, HBD < 5, and clog *P* < 5).^9^ Negligible differences are seen between class distributions, in which slightly higher values of HBA, clog *P*, and TPSA for the negative class may indicate that inaccurate GSE predictions might occur for bigger and more hydrophobic molecules. Moreover, the negative class presents slightly smaller solubility values. However, these differences are too small to acquire significant conclusions. A PCA using the 101 chosen descriptors was conducted (Figure 2b), revealing that PC1 and PC2 together account for 22.9% of the data set’s total variance. This finding indicates that unsupervised linear dimensionality reduction does not effectively differentiate between classes. However, the most contributing vectors for PC1 and PC2 showed a coinciding trend with the reappearance of the TPSA descriptor. Additionally, a nonlinear dimension reduction was also performed through *t*-SNE (Figure 2c). The clusters were marginal, in which the positive class (blue) tends to locate at the centroid of the map, and the negative classes were mostly positioned in the peripheries. Although both the PCA and the *t*-SNE showed insufficient capacity to cluster both classes, the *t*-SNE, although marginal, showed that the selected descriptors can slightly differentiate between the molecules with accurate and inaccurate GSE calculations. Figure 2d,e shows the most common fragments, obtained with the BRICS.decompose algorithm from RDKit (script available in the GitHub repository). Both classes present notable structural similarities, which might be attributed to the SolCbio3Database’s composition of mostly drug-like molecules. However, the positive class showed slightly higher frequencies of aromatic groups, while the negative class contained a higher number of amino groups. Differential analysis (Figure 2f) shows some statistically significant fragment differences between the molecules of both classes. Ether and sulfonated groups have higher representations within the negative class, whereas the positive class displays more halogenated aromatic rings and pyrimidine-based groups. However, although statistically significant, these structural divergences are marginal and not conclusive in determining the GSE viability, as Figure 2f evidence that these fragment frequencies lie between 1 and 8% differences. Therefore, a multivariate analysis, consisting of a hybrid ML and reinforcement-learning classification model based on chemoinformatic descriptors and structural information, might provide valuable information on the viability of the GSE in the SolCbio3 Database.

**Figure 2:**
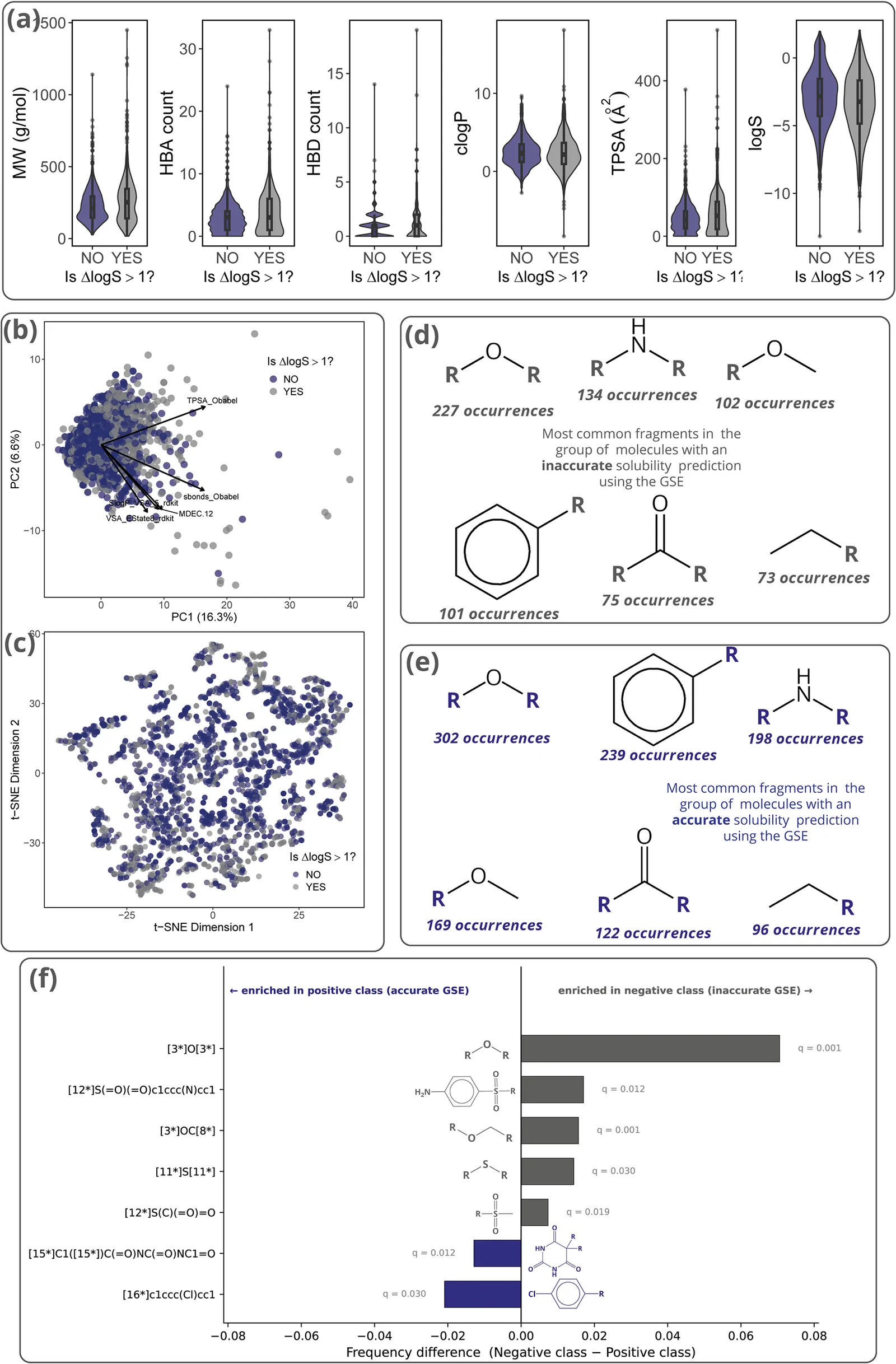
(a) Distribution of Lipinski’s rule of 5 physicochemical properties for both classes of molecules in the SolCbio3 Database (MW: molecular weight (g/mol), HBA/HBD: hydrogen bond acceptors/donors, clog *P*: calculated log* P*_*N*_ values using the Alogp algorithm, TPSA: topological polar surface area, and log *S*: experimental intrinsic solubility value). (b) PCA analysis of the database using the 101 significant descriptors via *t*-test. (c) *t*-SNE dimension reduction of the database using the 101 significant descriptors. (d) Most common structural fragments from the negative class (Δlog *S* > 1). (e) Most common structural fragments from the positive class (Δlog *S* ≤ 1). In every plot, the information on the positive and negative classes is labeled in blue and gray, respectively. (f) Frequency difference of fragments represented in each class enriched through a differential frequency analysis (two-tailed Fisher’s exact test with Benjamini–Hochberg corrections; *q* < 0.05).

### 3.2 Development of Interpretable Machine Learning Models

To address the challenge of performing effective clustering during chemical space visual exploration, molecular fingerprints were calculated and subsequently added to the descriptor list. The hybridization of 1D–4D chemoinformatic descriptors and molecular fingerprints is a commonly reported practice for the development of ML models for physicochemical and toxicological properties.^98,99^ The addition of 1024 binary variables into the data set brings issues of potential overfitted models.^100^ Therefore, an RFE algorithm was performed, which showed that 75 selected descriptors showed minimum error (Table S2).

Data sampling was conducted using simultaneous training/validation/test set division and 10-fold cross-validation. Table S3 presents the classification outcomes for each model, with key results summarized in Figure 3a as model accuracy, sensitivity, specificity, (eqs 10–11 and 13–15). The ANN, SVM, and NB models achieved promising accuracy levels. However, their lower specificity scores indicate challenges in correctly identifying negative class molecules within the test set. Despite high *F*_1_ scores, the specificity deficiencies were captured by the nMCC scores, which show values below 0.7. In contrast, while the RFC and XGBoost models exhibited similar accuracies, their higher specificity demonstrated greater efficacy in distinguishing between both classes, which was reflected mainly in their nMCC scores of ≥0.7. To assess potential improvements, an ensemble RFC + XGBoost model, based on soft voting predictions, was evaluated to determine if this combination would yield a more robust approach. Although accuracy and nMCC show improvements, a slight decrease in sensitivity was also observed compared to the independent RFC and XGBoost models.

**Figure 3:**
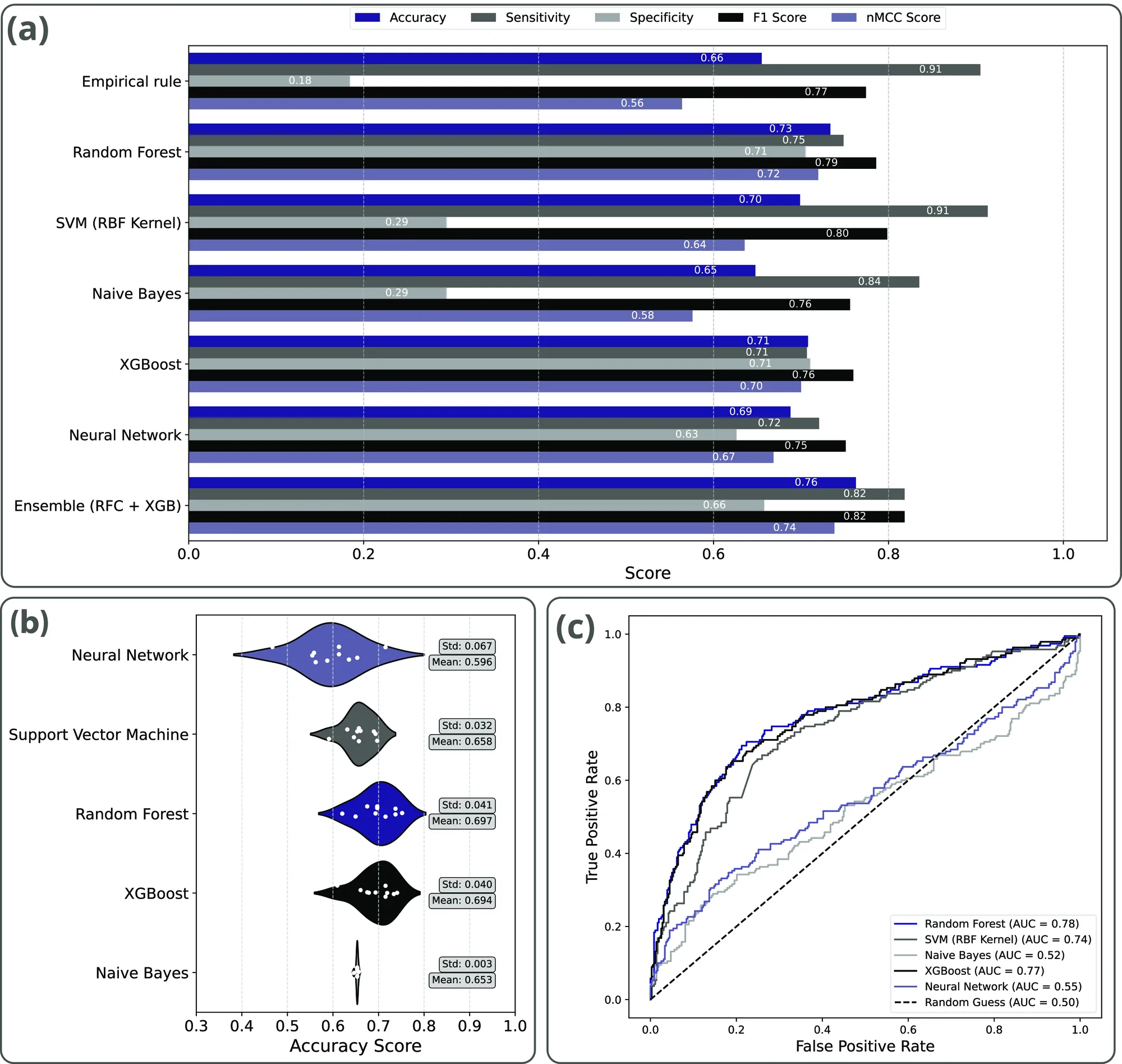
ML models’ performance in determining the viability of the GSE in the SolCbio3 Database test set. (a) Accuracy, sensitivity, specificity, *F*_1_, and nMCC values for the classification models, along with an average value of the two best models (RFC and ANN). (b) 10-fold CV accuracy distribution values for each model, in which the average and standard deviation of each distribution are reported. (c) ROC–AUC curves of each trained model, along with their respective AUC scores.

In addition, a rule-based decision model was established, based on an iterative evaluation of the SolCbio3Database’s mp and TPSA values between the positive and negative classes, motivated by the impact of these variables in the GSE.^53^ Figure S5 shows the distributions of these descriptors, along the entire database, for which the Youden’s *J*-based algorithm determined the optimal cutoff values between classes of mp = 142 °C and TPSA = 91.93 Å^2^, meaning that molecules with both values above this threshold will be labeled in the negative class. Figure 3a shows specificity deficiencies for this empirical decision model, which got outperformed by the RFC by 53% in specificity, and its nMCC score indicates predictions close to random guesses. This experiment demonstrates that the combination of 75 descriptors and molecular fingerprints captures molecular complexity that a two-variable heuristic cannot replicate. This comparison underscores the added value of the machine learning approach beyond straightforward descriptor thresholds.

Figure 3b illustrates the CV results, showing that the RFC achieved stronger training performance. This was indicated by the highest average accuracy, along with the smallest variation between iterations (0.70 ± 0.04). As a result, the RFC demonstrated superior predictive ability for distinguishing classes across a wider chemical space in the data set. Moreover, Figure 3c shows the ROC–AUC curves of each model. RFC and XGBoost models evidenced the highest AUC values (0.78 and 0.77, respectively), which indicate a higher diagnostic capability compared with the rest of the models. Based on these findings, the RFC was chosen (instead of XGBoost, due to similar performance and lower computational cost) to analyze the interpretability of the descriptors and to be implemented in a FAIR publicly accessible program.

A SHAP analysis was performed to assess the impact of individual descriptors on model decision-making. Figure 4a displays the Shapley scores for each data point across the 15 most influential descriptors. The melting point computed via OpenBabel (MP_Obabel) emerges as the primary contributor to the RFC’s predictions. Molecules exhibiting higher MP_Obabel values received greater Shapley scores, suggesting these compounds are more likely to yield accurate calculations with the GSE. As melting point is governed by structural features such as intermolecular interactions, stronger forces result in increased temperatures required for phase transitions. Additionally, TPSA (calculated with OpenBabel as TPSA_Obabel) demonstrated substantial influence. Here, molecules with lower TPSA_Obabel values were associated with favorable GSE outcomes. The significance of these two descriptors is noteworthy: melting point serves as a core parameter in the GSE (eq 1), while TPSA provides an empirical adjustment that enhances predictive performance.^53^ Figure 4b further underscores their importance by illustrating the RFC’s output decision-making process, where both MP_Obabel and TPSA_Obabel are prominent contributors to the predictions of the positive and negative classes.

**Figure 4:**
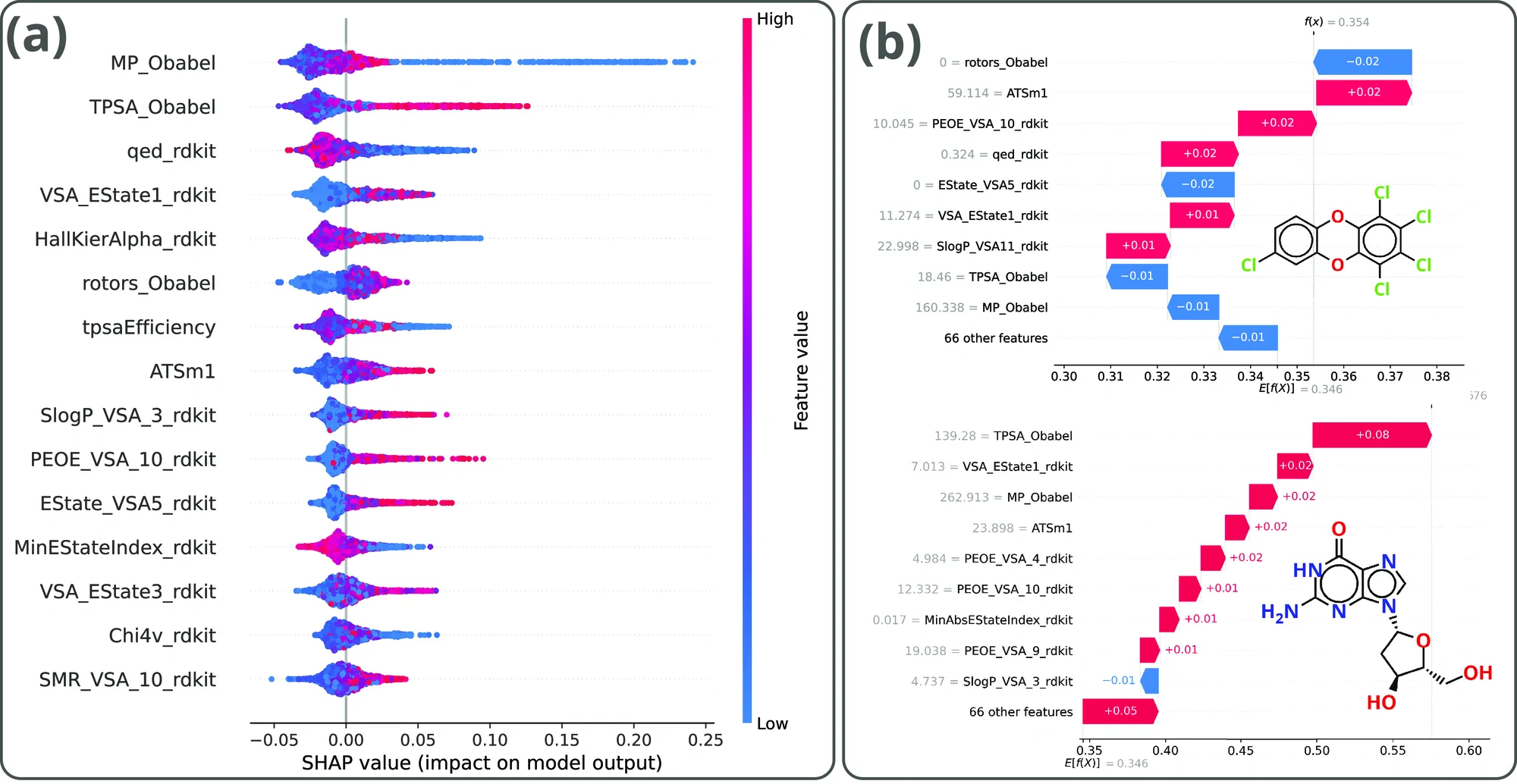
SHAP analysis of the most contributing descriptors to address the viability of the GSE in molecules through the RFC. (a) Summary plot of the top 15 most relevant descriptors. The color of each data point indicates the magnitude of the normalized value of each descriptor of the training set (blue = low value, red = high value). (b) Waterfall plot that indicates the decision pathway (blue = contribution to the positive class, red = contribution to the negative class) of the top 9 most contributing descriptors used for the classification of the entries for 1,2,3,4,7-pentachlorodibenzo-*p*-dioxin (top, positive class) and deoxyguanosine (bottom, negative class).

SHAP values also highlight similar tendencies in surface area characteristics through various RDKit descriptors that include the VSA acronym (van der Waals surface area), which represent the sum of atomic van der Waals surface area contributions within specific ranges given by other atomic descriptors.^101^ For instance, *S *log *P* and SMR indicate the sum of VSA of a specified range of atoms of the molecule of interest, given by calculated atomic log *P*_*N*_ and molar refraction, respectively.^84^ Similarly, Partial Equalization of Orbital Electronegativities (PEOE)^86^ and Electro-topological State (Estate)^85^ VSA descriptors give similar surface contributions based on these other charge-based and dipole moment atomic characteristics. Additionally, the ATSm1, an RCDK-based descriptor, consists of an autocorrelation topological descriptor based on the sum of atomic mass multiplication of neighboring atoms across the molecule, which means that ATSm1 correlates to the size of the molecule.^102^ This tendency is also highlighted with the Chi4v descriptor from RDKit, where its value correlates with the number of 4-atom pathways within a molecular graph.^103^ Figure 4 shows common trends for the VSA, ATSm1, and Chi4v descriptors, where smaller molecule size gets negative Shapley values, which indicates accurate GSE predictions. Moreover, the rotatable bonds count (rotors_obabel) was also labeled as relevant, where smaller counts indicate a better fit into the positive class (Figure 4a). This observation also correlates with the tendency of smaller molecules fitting better in the positive class. However, this descriptor also indicates that a higher rigidity might indicate a better possibility of having a good GSE prediction, as evidenced by 1,2,3,4,7-pentachlorodibenzo-*p*-dioxin in Figure 4b (top). Furthermore, tpsaEfficiency from RCDK consists of a relation of the TPSA of a molecule in terms of its molecular weight (TPSA/MW).^104^ This descriptor provides information on molecular size and the presence of polar heteroatoms of each molecule. Therefore, Shapley values in Figure 4a indicate that smaller and moderately polar molecules (higher tpsaEfficiency values, and validated with high MinEStateIndex_rdkit values, which correlate with the presence of dipole moments in a molecule^105^) favor viable GSE predictions. Moreover, HallKierAlpha from RDKit represents the sum of relative atomic size compared to the sp^3^ carbon.^103^ Shapely values indicate that higher HallKierAlpha values favor positive predictions, which indicate the presence of heavy heteroatoms in the positive class from higher periods, such as halogens, sulfur, or phosphorus. The presence of these atoms might indicate the presence of heavy molecules in the positive class and thus might contradict the findings from the TPSA-based descriptors. However, molecules like 1,2,3,4,7-pentachlorodibenzo-*p*-dioxin (Figure 4b, top), which are highly chlorinated molecules (and thus have a high HallKierAlpha value), maintain a relatively small surface area. Finally, the QED descriptor from RDKit—characterized as a drug-likeness score calculation based on desirability scores based on descriptors from Lipinski’s and Veber’s rules^106^—was labeled as the third most relevant descriptor. Higher QED values correlate with good GSE predictions within the data set. Despite the GSE being derived from a context of general un-ionized organic molecules, this finding indicates that Ro5-compliant molecules (which correlate with high QED scores^106^) provide adequate chemical characteristics to provide good GSE estimations.

In summary, the decision criteria of the RFC, as evidenced by SHAP analysis, suggest that smaller, rigid, polar, and drug-like molecules, characterized by high melting points, are more likely to yield accurate predictions when using the GSE. These molecular characteristics can be associated with physicochemical properties such as lipophilicity. Therefore, we examined the distribution of experimental log* P*_*N*_ values within our data set, and applied Youden’s *J*-statistic iterative method to determine the optimal log *P*_*N*_ threshold between classes (Figure S5, bottom). This analysis indicated that molecules over 5.42 log* P*_*N*_ units—characterized by nonpolar compounds—are more likely to provide unreliable GSE estimations. However, this examination is nonconclusive, since both classes contain molecules within the entire log* P*_*N*_ range in the SolCBio3Database [−7.4; 11.6], which further reinforces that multiple chemical variables are needed to meticulously describe the GSE variability.

### 3.3 GSESolver: FAIR Use and Practical Applications

The Google Colab notebook for GSESolver was developed to enhance user accessibility to the ANN, enabling users to assess the applicability of GSE for their molecules of interest. This notebook aligns with the FAIR principles according to the following criteria:^59^

1. Findable: GSESolver is readily discoverable and efficiently deployable. Users can access the Google Colab link directly from this paper, and an additional link is provided via the GitHub repository. The training data are openly available and can be located through both GitHub and Zenodo.^73^ Each data set entry references its experimental origin.
2. Accessible: Implementation in Google Colab facilitates broad accessibility, requiring only a Google account. The public GitHub repository further enables replication and script access via the “!git clone” command in the terminal. These tools support open, free, and straightforward implementation. Moreover, the repository’s public status allows users to submit comments and feedback, which may be incorporated into future updates, thus supporting continuous code improvement.
3. Interoperable: The Google Colab platform permits multiple users to execute scripts concurrently without compromising the original infrastructure. GSESolver is designed for ease of use, requiring minimal input, where users simply add their molecules as SMILES codes either directly in the notebook or via an Excel file. Comprehensive instructions accompany each step, which lowers programming barriers so that individuals with limited coding experience can successfully operate the models.
4. Reusable: Colab’s link-sharing feature allows users to modify the code as necessary while ensuring restoration of the original script upon page refresh, which ensures its reusability. GSESolver delivers results for small molecule data sets within approximately 5–10 min, with processing time increasing for larger data sets. Once an output file is generated, users can reuse the notebook by submitting new molecules or restarting the workflow. For enhanced efficiency, Colab’s licensing plans offer expanded computational resources, including faster GPUs and cloud storage. Alternatively, GSESolver can be used locally, where package installations are substantially faster compared to Colab servers.

We acknowledge that the GSE itself requires experimentally measured mp and log *P*_*N*_ to calculate log* S*_0_. However, the use case for GSESolver differs, which consists of predictions, from the SMILES string alone to assess whether the GSE is likely to provide an accurate prediction for a given molecule. In prospective screening scenarios, the necessary mp and log *P*_*N*_ values may be obtained from databases such as PubChem, DrugBank, or ChEMBL, or alternatively from high-throughput experimental measurements that are routinely performed in early drug discovery. For novel compounds not yet in databases, calculated log *P*_*N*_ values (e.g., Alog *P*,^107^ Crippen log *P*,^84^ milog *P* ^108^) and predicted mp (e.g., from OpenBabel^39^ or specialized QSPR^109^ or ML^46,110^ models) can serve as inputs, accepting that calculated parameters introduce additional uncertainty.

Two practical applications of GSESolver were evaluated to demonstrate its versatility. The first involved drug design and development, an area where solubility is crucial for determining oral bioavailability. In this case, GSESolver was applied to perform *in silico* estimations of a drug’s Maximum Absorbable Dose (MAD, eq 7).

Table 1 displays GSESolver’s predictions for the evaluated drugs, as well as the GSE-calculated MAD (eqs 1 and 7). Multiple errors in MAD calculation were noted, mainly because experimental solubilities were determined at a pH of 6.5, which indicates the possible presence of ionic species, whereas the GSE derivation was based on neutral solute states.^60^ This misalignment was mitigated by using the log* D*_6.5_ of the molecules, instead of their log* P*_*N*_. These log* D*_6.5_ values were computed using thermodynamic pH-dependent lipophilicity equations with the aid of the online tool LiProS, which indicates the most appropriate formalism for each molecule (Table S4).^76^ Despite the conceptual divergences, GSESolver’s predictions proved promising. As shown in Table 1, GSESolver successfully identified the most inaccurate MAD estimations, specifically for Azithromycin and Vardenafil. Both compounds had the highest error rates, but GSESolver recognized these cases, likely due to their elevated TPSA values: Azithromycin has a TPSA of 180 Å^2^ (greater than 98.5% of the SolCbio3Database), while Vardenafil has a TPSA of 118 Å^2^ (greater than 92.5% of the SolCbio3Database).

**Table 1:** Application of GSESolver Performing an *In Silico* Assessment of the MAD of a Small Set of Drugs^a^

drug^(ref)^	experimental MAD (mg)	GSESolver prediction	GSE MAD	ΔMAD^b^
**Azithromycin** ^10^	**34.02**	**“Do not use GSE”**	**4.7 × 10^4^**	**4.7 × 10^4^**
Rizatriptan^75^	0.9	“Use GSE”	17.1	16.2
**Vardenafil** ^75^	**0.1**	**“Do not use GSE”**	**417**	**417**
Propranolol^75^	7.7	“Use GSE”	5.2	2.5
Verapamil^75^	4.0	“Use GSE”	2.8	1.2
Nicotine^75^	3.4	“Use GSE”	134	130

a The two negative predictions were highlighted.

b ΔMAD = |MAD_exp_ – MAD_GSE_|.

The second practical application was designed as an intentional out-of-domain stress test, which involves using GSESolver in agrochemical design, where solubility is a key factor in pesticide effectiveness.^12,111^ Given that the SolCbio3Database is composed predominantly of drug-like molecules (Ro5-compliant, Figure 2a), a predictive performance in GSESolver might be anticipated when applied to agrochemicals, which occupy a substantially different region of chemical space. The explicit goal of this experiment consists of: (1) defining the boundaries of GSESolver’s applicability domain empirically, (2) identifying structural features responsible for performance degradation through, and (3) providing clear guidance to users about the chemical space in which the tool should and should not be applied. This approach is consistent with OECD guideline principles for QSAR model validation, which explicitly require characterization of the model’s domain of applicability.^112^ Therefore, the molecules reported by Zhang et al. were evaluated with GSE to assess GSESolver’s accuracy, sensitivity, and specificity for this purpose (eqs 10–12).

Figure 5a displays both the GSE-estimated and experimental values for the molecules tested. The GSE often miscalculated the intrinsic solubilities of various postemergency herbicides (PEHS) and chewing pest insecticides (CPIS). Figure 5b illustrates that GSESolver was effective in identifying molecules with the greatest inaccuracies in GSE estimation, whether residuals were positive or negative. Despite this, GSESolver’s overall accuracy was only 48% (Figure 5a), indicating many misclassifications by the model indistinguishable from random classification. Additionally, Figure 5b highlights a notable group of fungicides (FUNG, green dots) and sap-feeding pest insecticides (SFPI, yellow dots) displaying high GSE deviations, which GSESolver classified as “Use GSE”. As a result, the chemical characteristics of these pesticides were compared to the SolCbio3 Database molecules. Figure 5c shows the Lipinski distribution for the tested compounds, revealing that CPIS and PEHS—the molecules with the least accurate GSE predictions—had the largest molar mass and TPSA values, aligning with the previous SHAP analysis of GSE usage. Generally, most agrochemicals have higher HBA and HBD counts than those found in our database, while MAD drugs (black dots) are closer to the database average. In contrast, clog *P* and log* S* values indicate that agrochemicals are typically more hydrophobic and less soluble than SolCbio3 Database molecules, a trend commonly observed in pesticides. Empirical observations suggest that log *P*_*N*_ > 2 values often point to favorable conditions for bioaccumulation.^113,114^

**Figure 5:**
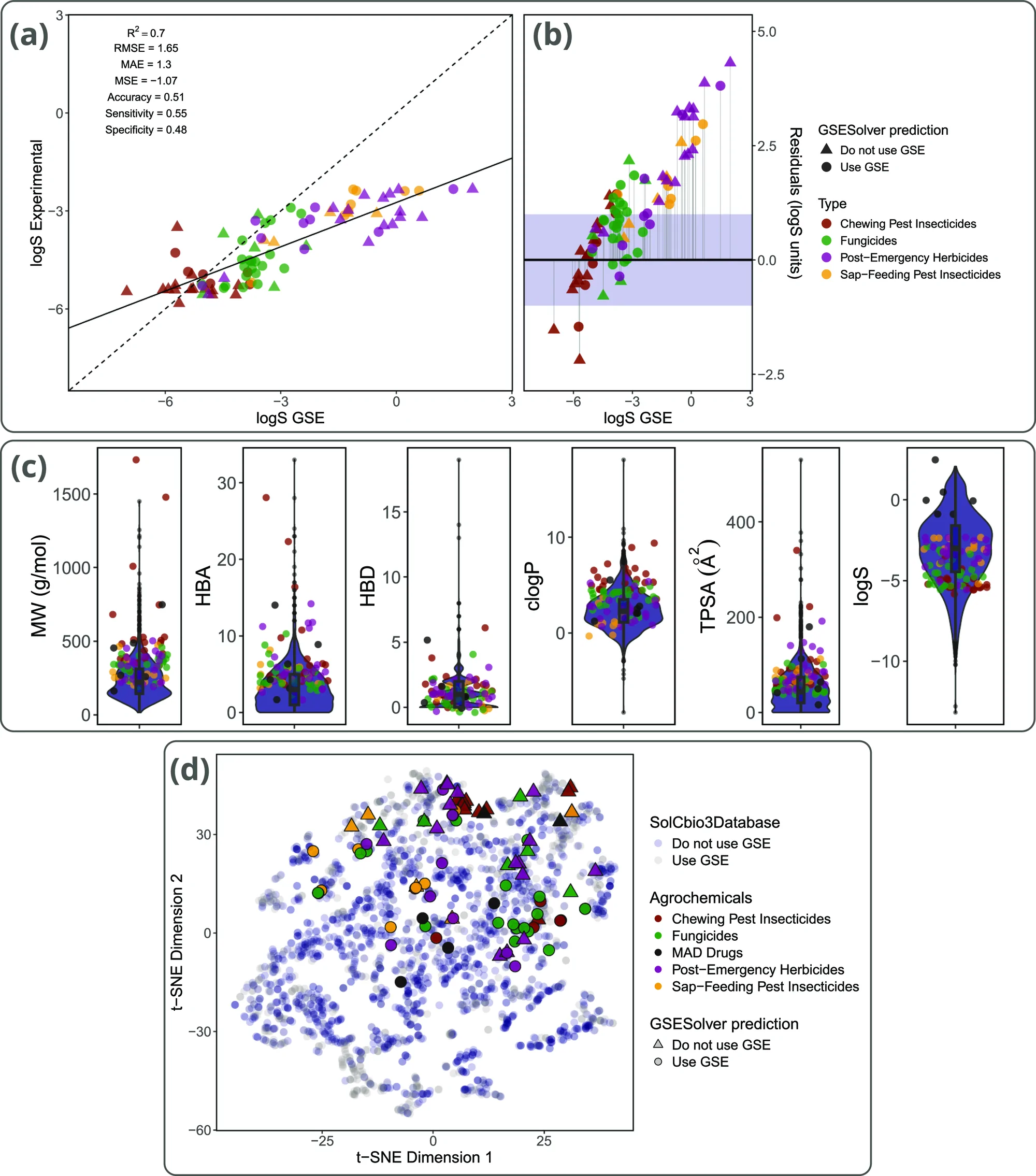
GSESolver predictions applied to the solubility of agrochemicals: (a) Correlation of the experimental and GSE-estimated solubilities. (b) Residual plot of the GSE estimations. The colors indicate the agrochemical type and the shapes indicate the GSESolver prediction. (c) (Blue Violins) Descriptor distribution of the SolCbio3 Database and (colored dots) the respective values of the tested agrochemical molecules and (black dots) drugs used for MAD estimations. The selected descriptors were chosen based on Lipinski’s rule of 5. (d) *t*-SNE projection of the agrochemicals and MAD drugs in contrast to the SolCbio3 Database.

Figure 5d presents a *t*-SNE visualization of GSESolver superposed onto the SolCbio3 Database, using the same 101 descriptors and hyperparameters. Positive predictions clustered at the map’s center, while negative ones appeared on the edges. On the right, many FUNG molecules fell into an “inaccurate GSE” zone on the map but were classified as positive. Similar inconsistencies are seen in the top left with FUNG and SFPI compounds. These results suggest that some chemical information is missing from the current descriptor set, likely due to absent fragments in the SolCbio3 Database, which may cause GSESolver to lose predictive power in this chemical space of pesticides. The “BRICS.decompose” algorithm was employed to examine the predominant fragments within these compounds. As illustrated in Figure 6, all groups exhibit a notably high frequency of halogenated fragments. Additionally, SFPI and FUNG compounds demonstrate substantial occurrences of imidazole derivatives. Both structural classes are underrepresented in the training data set, which may lead to inaccuracies by GSESolver when applied to this particular chemical space.

**Figure 6:**
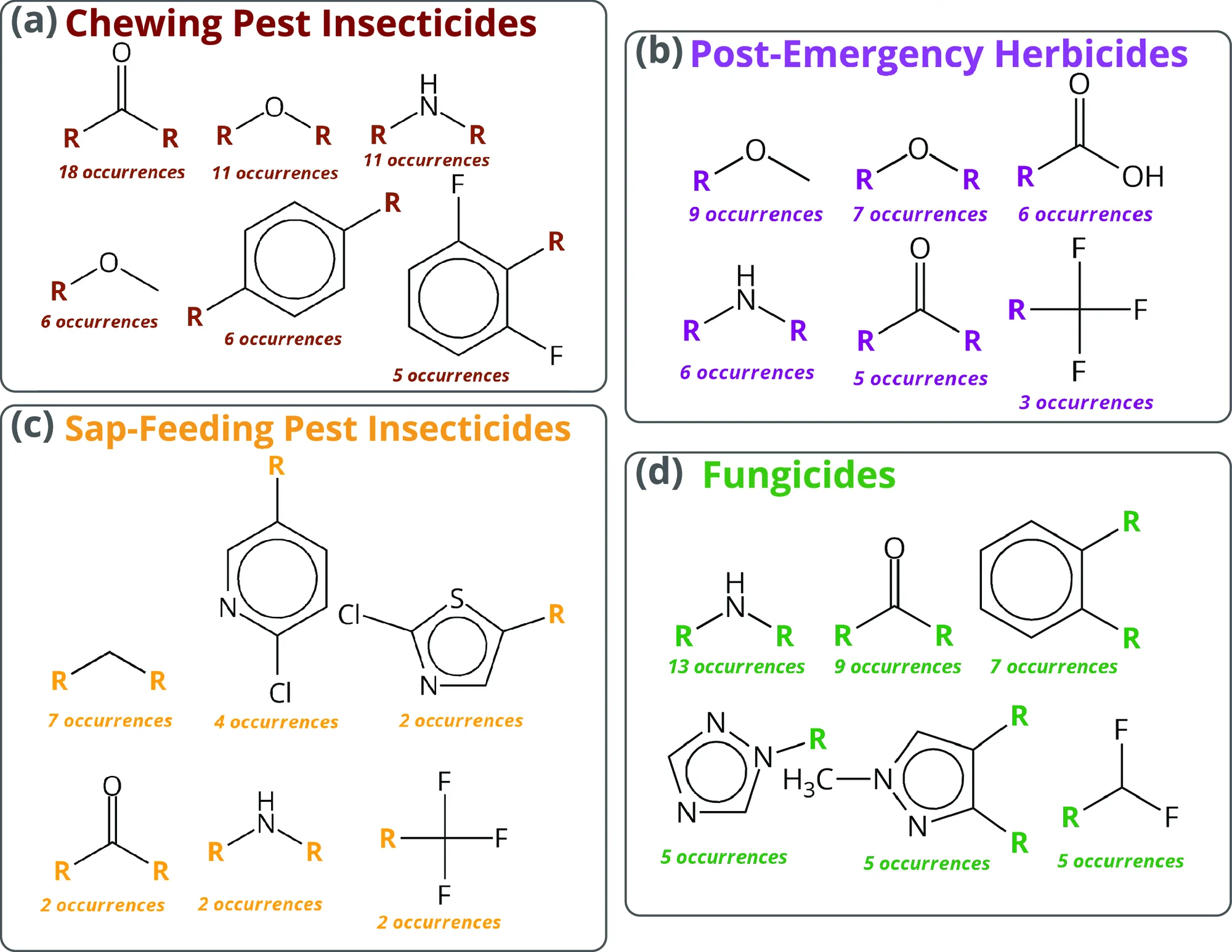
Most common fragments of the analyzed pesticides using GSESolver: (a) CPIS, (b) PEHS, (c) SFPI, and (d) FUNG compounds.

Vazquez-Salazar et al. highlighted that the chemical diversity within training data plays a key role in an ML model’s accuracy.^58^ The practical uses of GSESolver in this paper demonstrate the importance of acknowledging the chemical diversity on which GSESolver was trained. The inclusion of this agrochemical test case in the present study demonstrates the importance of applicability domain assessment before deploying any ML model to a novel chemical space, consistent with OECD guidelines for QSAR model validation.^112^ Alongside extensive discussion in this work, a disclaimer has also been added to the GSESolver Colab Notebook stating:

WARNING:

This model was trained primarily on small, drug-like (Ro5-compliant) organic molecules. Results may be unreliable for:

- Large molecules (MW > 1000 Da).
- Salts and organometallic complexes.
- Highly halogenated compounds.
- Agrochemicals: In a prospective test on 87 agrochemicals, GSESolver achieved only ∼48% accuracy (near-random). Do NOT use this tool for agrochemical solubility prediction without independent validation. Please inspect the chemical space of your molecules using Figure 5 before interpreting results.

## 4 Conclusions

The results of this large-scale evaluation demonstrate that the applicability of the general solubility equation (GSE) is strongly dependent on specific molecular characteristics. Descriptors, such as melting point and topological polar surface area (TPSA), emerged as key determinants of predictive reliability. Molecules with higher melting points tend to yield more accurate GSE predictions, likely reflecting stronger intermolecular interactions that align better with the thermodynamic assumptions of the model. Conversely, lower TPSA values (indicative of reduced polarity and simpler intermolecular interactions) are also associated with an improved performance. Additionally, trends observed in size-related descriptors suggest that smaller, less complex molecules are better suited for GSE-based predictions. Taken together, these findings establish that the GSE is most appropriate for relatively small, rigid, and moderately polar drug-like molecules with high melting points, while deviations from this chemical space increase the likelihood of significant prediction error.

The practical application of these findings was validated through case studies in medicinal chemistry, particularly in the estimation of the maximum absorbable dose (MAD). In this context, the GSE (when applied within its domain of validity) proved to be a useful and computationally efficient tool for early-stage drug development. However, its performance was notably less reliable in the agrochemical data set. This discrepancy can be attributed to the broader and more diverse chemical space of agrochemicals, which often includes larger, more flexible, and more polar compounds that fall outside the optimal applicability domain of GSE. These results highlight that while the GSE retains value in medicinal chemistry, its direct transferability to other domains such as agrochemical design is limited without additional corrections or alternative modeling strategies.

Finally, the development of GSESolver represents a significant advancement for the medicinal chemistry community by addressing a critical gap in the practical use of GSE. Rather than replacing the equation, GSESolver enhances its utility by enabling users to rapidly assess, from the molecular structure alone, whether the GSE is likely to provide reliable solubility predictions. Built under FAIR principles and implemented in an accessible Google Colab environment, the tool lowers technical barriers and promotes reproducibility and transparency. Its integration of machine learning with physicochemical insight allows researchers to make informed decisions during early drug design, reducing the risk of misapplication of the GSE and improving the efficiency in compound prioritization. As such, GSESolver serves as a valuable, user-friendly decision support tool that complements existing solubility prediction methodologies.

## Supplementary Material



## Data Availability

The curated data set with experimental lipophilicity values can be found in ref 73. Further data sets, descriptors, scripts, and model training can be found in the repository https://github.com/cbio3lab/GSESolver.git. The Google Colab Notebook can be freely used with this link: https://colab.research.google.com/drive/1bzTD7NvdyLNfh4UF2GJ0UN-swyYTDD4P?usp=sharing.

## Notes

Distribution of the standard deviations of solubility values of 172 compounds from the SolCbio3 Database with overlapping entries across two or more source databases (Figure S1); correlation coefficients between the selected descriptors after the Univariate Feature Selection (Figure S2); (left) distribution of Δlog *S* values after evaluating the GSE in the SolCbio3 Database. (*right*) Columns: Number of data entries that indicate a GSE prediction where Δlog* S* ≤ *i* (left vertical axis). Points: Number of descriptors for each tested Δlog* S* threshold that showed significant separation between groups through the UFS (Figure S3); distribution of log* P*_*N*_ and mp values in function of Δlog *S*, from comparing experimental and GSE-estimated intrinsic solubility values in the SolCbio3 Database (Figure S4); density plots that integrate the optimal decision thresholds for an empirical rule-based model for the GSE viability based on the melting point (top left), TPSA (top right), and log *P*_*N*_ (bottom) of the SolCbio3 Database after an iterative Youden’s *J*-statistic algorithm (optimal values: mp = 142 °C, TPSA = 91.93 Å^2^, log *P_N_* = 5.42) (Figure S5); selected descriptors after the Univariate Feature Selection performed by performing a two-tailed Welch’s *t* test. Documentation for each descriptor is provided (Table S1); selected descriptors to train the classification ML models after the Recursive Feature Elimination (Table S2); classification results for each ML model (Table S3); data used to estimate the MAD of a selected set of drugs, in which GSESolver was tested (Table S4); experimental, computed, and GSE-predicted data of a small set of agrochemicals, in which GSESolver was tested (Table S5). The following references were cited in the Supporting Information.^10,12,75,76,83–87,91,115,116^ (PDF)

## References

[1] The IUPAC Compendium of Chemical Terminology . Solubility (S05740). https://goldbook.iupac.org/terms/view/S05740.

[2] Llompart P., Minoletti C., Baybekov S. (2024). Will we ever be able
to accurately predict solubility?. Sci. Data.

[3] Saal C., Petereit A. C. (2012). Optimizing solubility:
Kinetic versus thermodynamic
solubility temptations and risks. Eur. J. Pharm.
Sci..

[4] Han Y. R., Lee P. I. (2017). Effect
of Extent of Supersaturation on the Evolution
of Kinetic Solubility Profiles. Mol. Pharmaceutics.

[5] Avdeef A. (2007). Solubility
of sparingly-soluble ionizable drugs. Adv. Drug
Delivery Rev..

[6] Wang J., Hou T. (2011). Recent Advances
on Aqueous Solubility Prediction. CCHTS.

[7] Zhao J., Hermans E., Sepassi K. (2025). Improved estimation
of intrinsic solubility of drug-like molecules through multi-task
graph transformer. J. Cheminf..

[8] Di L., Fish P. V., Mano T. (2012). Bridging solubility between drug
discovery and development. Drug Discovery Today.

[9] Lipinski C. A., Lombardo F., Dominy B. W., Feeney P. J. (2001). Experimental and
computational approaches to estimate solubility and permeability in
drug discovery and development settings. Adv.
Drug Delivery Rev..

[10] Curatolo W. J. (1998). Physical
chemical properties of oral drug candidates in the discovery and exploratory
development settings. Pharm. Sci. Technol. Today.

[11] Fiese E. F. (2003). “Gene”.
General pharmaceutics—The new physical pharmacy. J. Pharm. Sci..

[12] Zhang Y., Lorsbach B. A., Castetter S. (2018). Physicochemical property
guidelines for modern agrochemicals. Pest Manage.
Sci..

[13] Mulqueen P. (2003). Recent advances
in agrochemical formulation. Adv. Colloid Interface
Sci..

[14] Lucero-Borja D., Subirats X., Barbas R. (2020). Potentiometric CheqSol
and standardized shake-flask solubility methods are complimentary
tools in physicochemical profiling. Eur. J.
Pharm. Sci..

[15] Palmer D. S., Llinàs A., Morao I. (2008). Predicting Intrinsic
Aqueous Solubility by a Thermodynamic Cycle. Mol. Pharmaceutics.

[16] Gupta J., Nunes C., Vyas S., Jonnalagadda S. (2011). Prediction
of Solubility Parameters and Miscibility of Pharmaceutical Compounds
by Molecular Dynamics Simulations. J. Phys.
Chem. B.

[17] Liu S., Cao S., Hoang K. (2016). Using MD Simulations To Calculate How Solvents
Modulate Solubility. J. Chem. Theory Comput..

[18] Bjelobrk Z., Mendels D., Karmakar T., Parrinello M., Mazzotti M. (2021). Solubility Prediction of Organic
Molecules with Molecular
Dynamics Simulations. Cryst. Growth Des..

[19] Suarez A. G., Göller A. H., Beck M. E., Gheta S. K. O., Meier K. (2024). Comparative
assessment of physics-based in silico methods to calculate relative
solubilities. J. Comput.-Aided Mol. Des..

[20] Klamt A., Eckert F., Hornig M., Beck M. E., Bürger T. (2002). Prediction
of aqueous solubility of drugs and pesticides with COSMO-RS. J. Comput. Chem..

[21] Eckert F., Klamt A. (2002). Fast solvent screening via quantum chemistry: COSMO-RS approach. AIChE J..

[22] Mullins E., Liu Y. A., Ghaderi A., Fast S. D. (2008). Sigma Profile Database
for Predicting Solid Solubility in Pure and Mixed Solvent Mixtures
for Organic Pharmacological Compounds with COSMO-Based Thermodynamic
Methods. Ind. Eng. Chem. Res..

[23] Abramov Y. A., Sun G., Zeng Q., Zeng Q., Yang M. (2020). Guiding Lead Optimization
for Solubility Improvement with Physics-Based Modeling. Mol. Pharmaceutics.

[24] López-López E., Espinoza-Castañeda J. I., Martinez-Mayorga K., Medina-Franco J. L. (2026). Structure–Property Associations:
Breaking Paradigms
for Linking Chemical Structures and Biological Properties in Drug
Discovery. ChemMedChem.

[25] Dearden J. C. (2006). In silico
prediction of aqueous solubility. Expert Opin.
Drug Discovery.

[26] Huuskonen J. (2000). Estimation
of aqueous solubility for a diverse set of organic compounds based
on molecular topology. J. Chem. Inf. Comput.
Sci..

[27] McFarland J. W., Avdeef A., Berger C. M., Raevsky O. A. (2001). Estimating the water
solubilities of crystalline compounds from their chemical structures
alone. J. Chem. Inf. Comput. Sci..

[28] Jorgensen W. L., Duffy E. M. (2002). Prediction of drug
solubility from structure. Adv. Drug Delivery
Rev..

[29] Yan A., Gasteiger J. (2003). Prediction
of aqueous solubility of organic compounds
based on a 3D structure representation. J. Chem.
Inf. Comput. Sci..

[30] Cheng A., Cheng A., Merz K. M. (2003). Prediction of Aqueous Solubility
of a Diverse Set of Compounds Using Quantitative Structure–Property
Relationships. J. Med. Chem..

[31] Delaney J.
S. (2004). ESOL: Estimating
Aqueous Solubility Directly from Molecular Structure. J. Chem. Inf. Comput. Sci..

[32] Duchowicz P. R., Talevi A., Bruno-Blanch L. E., Castro E. A. (2008). New QSPR study for
the prediction of aqueous solubility of drug-like compounds. Bioorg. Med. Chem..

[33] Meftahi N., Walker M. L., Smith B. J. (2021). Predicting aqueous
solubility by
QSPR modeling. J. Mol. Graphics Modell..

[34] Sushko I., Pandey A., Novotarskyi S. (2011). Online chemical modeling
environment (OCHEM): web platform for data storage, model development
and publishing of chemical information. J. Cheminf..

[35] Sorkun M. C., Khetan A., Er S. (2019). AqSolDB, a curated
reference set
of aqueous solubility and 2D descriptors for a diverse set of compounds. Sci. Data.

[36] Krasnov L., Malikov D., Kiseleva M. (2025). BigSolDB 2.0, dataset
of solubility values for organic compounds in different solvents at
various temperatures. Sci. Data.

[37] RDKit. https://www.rdkit.org/.

[38] Willighagen, E. L. Groovy Cheminformatics with the Chemistry Development Kit (v2.8–1), 2023. https://egonw.github.io/cdkbook/.

[39] O’Boyle N. M., Banck M., James C. A. (2011). Open Babel: An open
chemical toolbox. J. Cheminf..

[40] Pedregosa F. (2011). Scikit-learn: Machine Learning in Python. J.
Mach. Learn. Res..

[41] Abadi, M. ; Agarwal, A. ; Barham, P. ; Brevdo, E. TensorFlow: Large-scale machine learning on heterogeneous systems, 2015. https://www.tensorflow.org/.

[42] Lusci A., Pollastri G., Baldi P. (2013). Deep Architectures and Deep Learning
in Chemoinformatics: The Prediction of Aqueous Solubility for Drug-Like
Molecules. J. Chem. Inf. Model..

[43] Cui Q., Lu S., Ni B. (2020). Improved Prediction of Aqueous Solubility of
Novel Compounds by Going Deeper With Deep Learning. Front. Oncol..

[44] Francoeur P. G., Koes D. R. (2021). SolTranNet–A
Machine Learning Tool for Fast
Aqueous Solubility Prediction. J. Chem. Inf.
Model..

[45] Lovrić M., Pavlović K., Žuvela P. (2021). Machine learning in
prediction of intrinsic aqueous solubility of drug-like compounds:
Generalization, complexity, or predictive ability?. J. Chemom..

[46] Zhu X., Polyakov V. R., Bajjuri K. (2023). Building Machine Learning
Small Molecule Melting Points and Solubility Models Using CCDC Melting
Points Dataset. J. Chem. Inf. Model..

[47] Bao Z., Tom G., Cheng A., Watchorn J., Aspuru-Guzik A., Allen C. (2024). Towards the Prediction
of Drug Solubility in Binary Solvent Mixtures
at Various Temperatures Using Machine Learning. J. Cheminform.

[48] Ramos M. C., White A. D. (2024). Predicting small
molecules solubility on endpoint devices
using deep ensemble neural networks. Digital
Discovery.

[49] Jain N., Yalkowsky S. H. (2001). Estimation
of the aqueous solubility I: Application
to organic nonelectrolytes. J. Pharm. Sci..

[50] Sanghvi T., Jain N., Yang G., Yalkowsky S. H. (2003). Estimation
of Aqueous Solubility By The General Solubility Equation (GSE) The
Easy Way. QSAR Comb. Sci..

[51] Yalkowsky S. H., Valvani S. C. (1980). Solubility and partitioning
I: Solubility of nonelectrolytes
in water. J. Pharm. Sci..

[52] Ran Y., He Y., Yang G., Johnson J. L. H., Yalkowsky S. H. (2002). Estimation
of aqueous solubility of organic compounds by using the general solubility
equation. Chemosphere.

[53] Ali J., Camilleri P., Brown M. B. (2012). Revisiting the General
Solubility Equation: In Silico Prediction of Aqueous Solubility Incorporating
the Effect of Topographical Polar Surface Area. J. Chem. Inf. Model..

[54] Stienstra C. M. K., Ieritano C., Haack A., Hopkins W. S. (2023). Bridging the Gap
between Differential Mobility, Log S, and Log P Using Machine Learning
and SHAP Analysis. Anal. Chem..

[55] Su M., Herrero E. (2023). Creation and interpretation
of machine learning models
for aqueous solubility prediction. Explor. Drug
Sci..

[56] Gao Y. (2026). Application
of high-precision solubility prediction models in the assisted design
of drug-like compounds. Mol. Diversity.

[57] Ghanavati M. A., Ahmadi S., Rohani S. (2024). A machine learning
approach for the
prediction of aqueous solubility of pharmaceuticals: a comparative
model and dataset analysis. Digital Discovery.

[58] Vazquez-Salazar L. I., Boittier E. D., Unke O. T., Meuwly M. (2021). Impact of the Characteristics
of Quantum Chemical Databases on Machine Learning Prediction of Tautomerization
Energies. J. Chem. Theory Comput..

[59] Wilkinson M. D., Dumontier M., Aalbersberg I. J. (2016). The FAIR Guiding Principles
for scientific data management and stewardship. Sci. Data.

[60] Ran Y., Yalkowsky S. H. (2001). Prediction
of Drug Solubility by the General Solubility
Equation (GSE). J. Chem. Inf. Comput. Sci..

[61] Shoghi E., Fuguet E., Bosch E., Ràfols C. (2013). Solubility–pH
profiles of some acidic, basic and amphoteric drugs. Eur. J. Pharm. Sci..

[62] Avdeef A., Kansy M. (2020). Flexible-Acceptor” General
Solubility Equation for beyond
Rule of 5 Drugs. Mol. Pharmaceutics.

[63] Avdeef A., Kansy M. (2020). Can small drugs predict
the intrinsic aqueous solubility of ‘beyond
Rule of 5′ big drugs?. ADMET DMPK.

[64] Avdeef, A. Predicting Solubility of New Drugs: Handbook of Critically Curated Data for Pharmaceutical Research; Taylor and Francis Group: Boca Ratón, Florida, 2024.

[65] Weininger D. (1988). SMILES, a
chemical language and information system. 1. Introduction to methodology
and encoding rules. J. Chem. Inf. Comput. Sci..

[66] Frolov A. I., Chankeshwara S. V., Abdulkarim Z., Ghiandoni G. M. (2023). pIChemiSt—Free
Tool for the Calculation of Isoelectric Points of Modified Peptides. J. Chem. Inf. Model..

[67] Introduction — MolVS 0.1.1 Documentation. https://molvs.readthedocs.io/en/latest/guide/intro.html.

[68] Palmer D. S., Mitchell J. B. O. (2014). Is Experimental Data Quality the
Limiting Factor in
Predicting the Aqueous Solubility of Druglike Molecules?. Mol. Pharmaceutics.

[69] Bergström C. A., Avdeef A. (2019). Perspectives in solubility measurement and interpretation. ADMET DMPK.

[70] Attia L., Burns J. W., Doyle P. S., Green W. H. (2025). Data-driven organic
solubility prediction at the limit of aleatoric uncertainty. Nat. Commun..

[71] Zamora Ramírez, W. J. ; Lopez Perez, K. n-Octanol/Water Partition Coefficient Database, Zenodo, 2026 10.5281/zenodo.18616059.

[72] Kim S., Chen J., Cheng T. (2025). PubChem 2025 update. Nucleic Acids Res..

[73] Bertsch-Aguilar, E. ; Schosinsky, F. ; Quiros, N. ; Pinheiro, S. ; Zamora Ramírez, W. J. CBio3 Laboratory Solubility Database: SolCbio3Database, Zenodo, 2026. 10.5281/zenodo.18624099.

[74] Johnson K. C., Swindell A. C. (1996). Guidance in the
Setting of Drug Particle Size Specifications
to Minimize Variability in Absorption. Pharm.
Res..

[75] Yang Z., Sotthivirat S., Wu Y. (2016). Application of *in vitro* transmucosal permeability, dose number, and maximum
absorbable dose for biopharmaceutics assessment during early drug
development for intraoral delivery. Int. J.
Pharm..

[76] Bertsch-Aguilar E., Piedra A., Acuña D. (2025). LiProS: Findable, Accessible,
Interoperable, and Reusable Data Simulation Workflow to Predict Accurate
Lipophilicity Profiles for Small Molecules. Mol. Inf..

[77] pKa Plugin|Chemaxon Docs. https://docs.chemaxon.com/display/docs/pka-plugin.md.

[78] Munoz-muriedas, J. Bioavailability Prediction at Early Drug Discovery Stages: *In Vitro* Assays and Simple Physico-Chemical Rules. In Physico-Chemical and Computational Approaches to Drug Discovery; Luque, J. ; Barril, X. , Eds.; The Royal Society of Chemistry, 2012; pp 104–127.

[79] Ghiandoni G. M., Caldeweyher E. (2023). Fast calculation of hydrogen-bond strengths and free
energy of hydration of small molecules. Sci.
Rep..

[80] AstraZeneca/jazzy, 2025. https://github.com/AstraZeneca/jazzy.

[81] Zamora W. J., Viayna A., Pinheiro S. (2023). Prediction of toluene/water
partition coefficients in the SAMPL9 blind challenge: assessment of
machine learning and IEF-PCM/MST continuum solvation models. Phys. Chem. Chem. Phys..

[82] Welch B. L. (1947). The Generalization
Of ‘Student’s’ Problem When Several Different
Population Variances Are Involved. Biometrika.

[83] Kier L. B. (1987). Indexes
of molecular shape from chemical graphs. Med.
Res. Rev..

[84] Wildman S. A., Crippen G. M. (1999). Prediction of Physicochemical Parameters by Atomic
Contributions. J. Chem. Inf. Comput. Sci..

[85] Hall L. H., Kier L. B. (1995). Electrotopological
State Indices for Atom Types: A
Novel Combination of Electronic, Topological, and Valence State Information. J. Chem. Inf. Comput. Sci..

[86] Gasteiger J., Marsili M. (1980). Iterative partial equalization
of orbital electronegativity—a
rapid access to atomic charges. Tetrahedron.

[87] Nguyen K. T., Blum L. C., van Deursen R., Reymond J.-L. (2009). Classification of
Organic Molecules by Molecular Quantum Numbers. ChemMedChem.

[88] Veber D. F., Johnson S. R., Cheng H. Y. (2002). Molecular properties
that influence the oral bioavailability of drug candidates. J. Med. Chem..

[89] Agresti A. (1992). A Survey of
Exact Inference for Contingency Tables. Stat.
Sci..

[90] Benjamini Y., Hochberg Y. (1995). Controlling the False
Discovery Rate: A Practical and
Powerful Approach to Multiple Testing. J. Royal
Stat. Soc., Ser. B: Stat. Methodol..

[91] scikit-learn: Machine Learning in Python — Scikit-learn 1.7.2 Documentation. https://scikit-learn.org/stable/.

[92] Youden W. J. (1950). Index for
rating diagnostic tests. Cancer.

[93] Chen, T. ; Guestrin, C. In XGBoost: A Scalable Tree Boosting System, Proceedings of the 22nd ACM SIGKDD International Conference on Knowledge Discovery and Data Mining; ACM: San Francisco CA, 2016; pp 785–794.

[94] Chicco D., Jurman G. (2023). The Matthews correlation
coefficient (MCC) should replace
the ROC AUC as the standard metric for assessing binary classification. BioData Min..

[95] Lundberg, S. M. ; Lee, S.-I. In A Unified Approach to Interpreting Model Predictions, Advances in Neural Information Processing Systems; Vol. 30, Curran Associates, Inc., 2017.

[96] Welcome to the SHAP Documentation—SHAP Latest Documentation. https://shap.readthedocs.io/en/latest/index.html.

[97] Kumar P., Bhatnagar R., Gaur K., Bhatnagar A. (2021). Classification
of Imbalanced Data:Review of Methods and Applications. IOP Conf. Ser.: Mater. Sci. Eng..

[98] Kim D., Jeong J., Choi J. (2024). Identification
of Optimal Machine
Learning Algorithms and Molecular Fingerprints for Explainable Toxicity
Prediction Models Using ToxCast/Tox21 Bioassay Data. ACS Omega.

[99] Fenogli, J. ; Grimaud, L. ; Vuilleumier, R. Constructing and Explaining Machine Learning Models for Chemistry: Example of the Exploration and Design of Boron-based Lewis Acids. 2025, arXiv:2501.01576. arXiv.org e-Print archive. https://arxiv.org/abs/2501.01576.

[100] Ying X. (2019). An Overview of Overfitting and its
Solutions. J. Phys.: Conf. Ser..

[101] El-Atawneh S., Goldblum A. (2023). Activity Models of
Key GPCR Families
in the Central Nervous System: A Tool for Many Purposes. J. Chem. Inf. Model..

[102] Hollas B. (2003). An Analysis of the Autocorrelation Descriptor for Molecules. J. Math. Chem..

[103] Hall, L. H. ; Kier, L. B. The Molecular Connectivity Chi Indexes and Kappa Shape Indexes in Structure-Property Modeling. In Reviews in Computational Chemistry; Lipkowitz, K. B. ; Boyd, D. B. , Eds.; Wiley, 1991; Vol. 2, pp 367–422.

[104] FractionalPSADescriptor (cdk 1.5.15 API). https://cdk.github.io/cdk/1.5/docs/api/org/openscience/cdk/qsar/descriptors/molecular/FractionalPSADescriptor.html.

[105] Hall L. H., Mohney B., Kier L. B. (1991). The electrotopological
state: structure information at the atomic level for molecular graphs. J. Chem. Inf. Comput. Sci..

[106] Bickerton G. R., Paolini G. V., Besnard J., Muresan S., Hopkins A. L. (2012). Quantifying the chemical beauty of drugs. Nat. Chem..

[107] Ghose A. K., Viswanadhan V. N., Wendoloski J. J. (1998). Prediction
of Hydrophobic (Lipophilic) Properties of Small Organic Molecules
Using Fragmental Methods: An Analysis of ALOGP and CLOGP Methods. J. Phys. Chem. A.

[108] logP - Octanol-Water Partition Coefficient Calculation. https://www.molinspiration.com/services/logp.html.

[109] Karthikeyan M., Glen R. C., Bender A. (2005). General Melting
Point
Prediction Based on a Diverse Compound Data Set and Artificial Neural
Networks. J. Chem. Inf. Model..

[110] Song S., Wang Y., Tian X. (2023). Predicting
the Melting Point of Energetic Molecules Using a Learnable Graph Neural
Fingerprint Model. J. Phys. Chem. A.

[111] Baker E. A., Hayes A. L., Butler R. C. (1992). Physicochemical
properties of agrochemicals: Their effects on foliar penetration. Pestic. Sci..

[112] OECD . Guidance Document on the Validation of (Quantitative) Structure-Activity Relationship [(Q)SAR] Models, OECD Series on Testing and Assessment, 2014.

[113] Gobas F. A. P. C., Kelly B. C., Arnot J. A. (2003). Quantitative
Structure
Activity Relationships for Predicting the Bioaccumulation of POPs
in Terrestrial Food-Webs. QSAR Comb. Sci..

[114] Lee Y.-S., Cole T. R., Jhutty M. S. (2022). Bioaccumulation
Screening of Neutral Hydrophobic Organic Chemicals in Air-Breathing
Organisms Using In Vitro Rat Liver S9 Biotransformation Assays. Environ. Toxicol. Chem..

[115] Shannon C. E. (1948). A Mathematical
Theory of Communication. Bell Syst. Tech. J..

[116] XGBoost Parameters — xgboost 3.1.1 Documentation. https://xgboost.readthedocs.io/en/stable/parameter.html.

