# Supplementary material for: Data-Driven Critical Evaluation of the General Solubility Equation
Source: J Chem Inf Model. 2026 Aug 24;66(17):11330–47. doi: 10.1021/acs.jcim.6c01182 (PMC13580124; doi:10.1021/acs.jcim.6c01182)
Supplement: Supplementary file 1 [file ci6c01182_si_001.pdf]

Supporting Information

Data-driven critical evaluation of the General Solubility Equation: When is it a valid solubility predictor for Drug-like Molecules?

Esteban Bertsch-Aguilar[1,2*], Nahomy Quirós[2], Frederick Schosinsky[2], Silvana Pinheiro[2], José L. Medina-Franco[3], William J. Zamora[1,2,4*]

[1]CBio^3 Laboratory, School of Chemistry, Universidad de Costa Rica, San Pedro, San José 11501, Costa Rica.
[2]Laboratory of Computational Toxicology and Artificial Intelligence (LaToxCIA), Biological Testing Laboratory (LEBi), Universidad de Costa Rica, San Pedro, San José 11501, Costa Rica.
[3]DIFACQUIM Research Group, Department of Pharmacy, School of Chemistry, Universidad Nacional Autónoma de México, Mexico City 04510, Mexico.
[4]National Advanced Computing Collaboratory (CNCA), National High Technology Center (CeNAT), Pavas, San José 11501, Costa Rica.

*Corresponding Authors (esteban.bertsch@ucr.ac.cr and william.zamoraramirez@ucr.ac.cr)

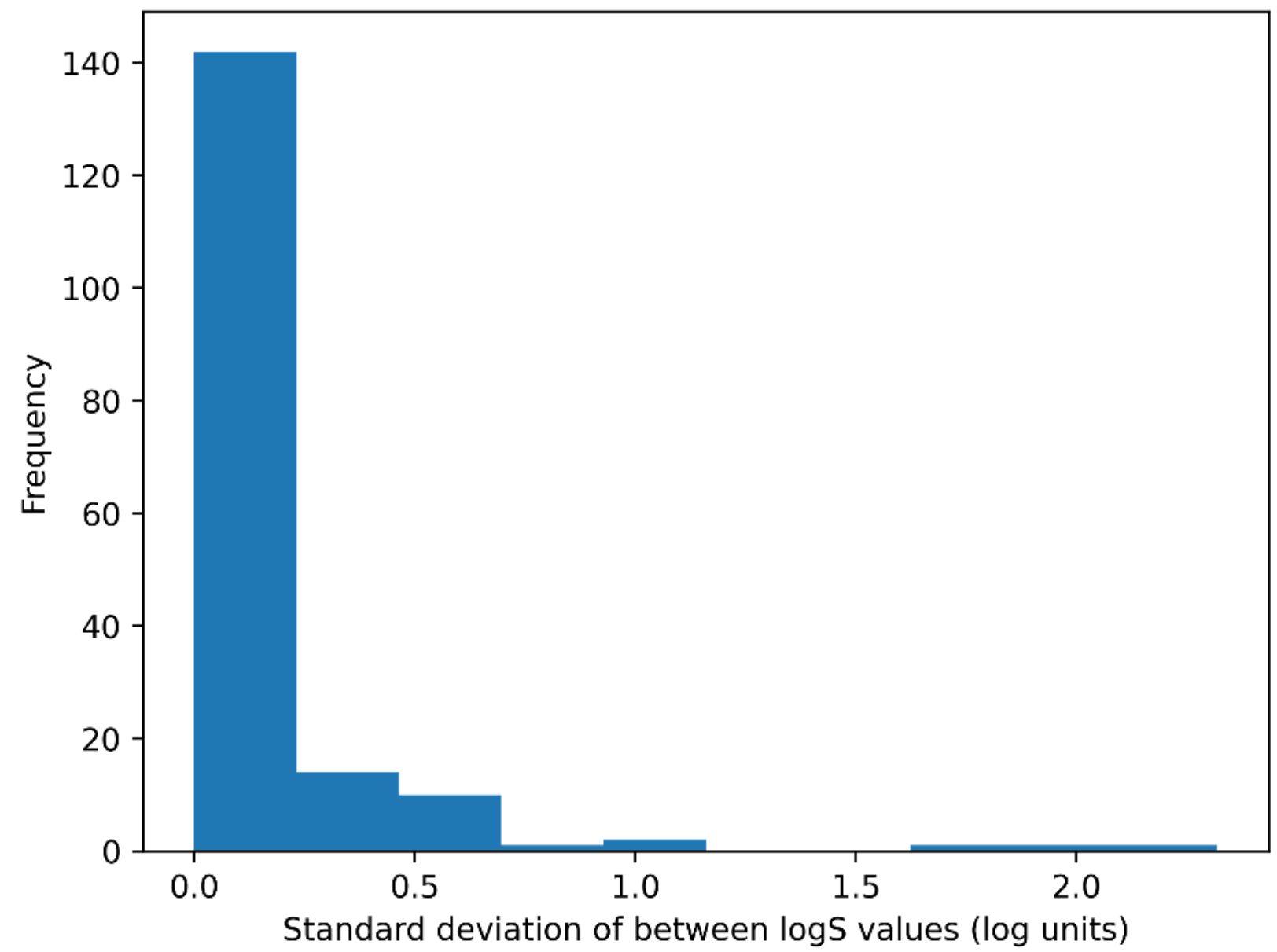

Figure S1. Distribution of the standard deviations of solubility values of 172 compounds from SolCbio3Database with overlapping entries across two or more source databases.

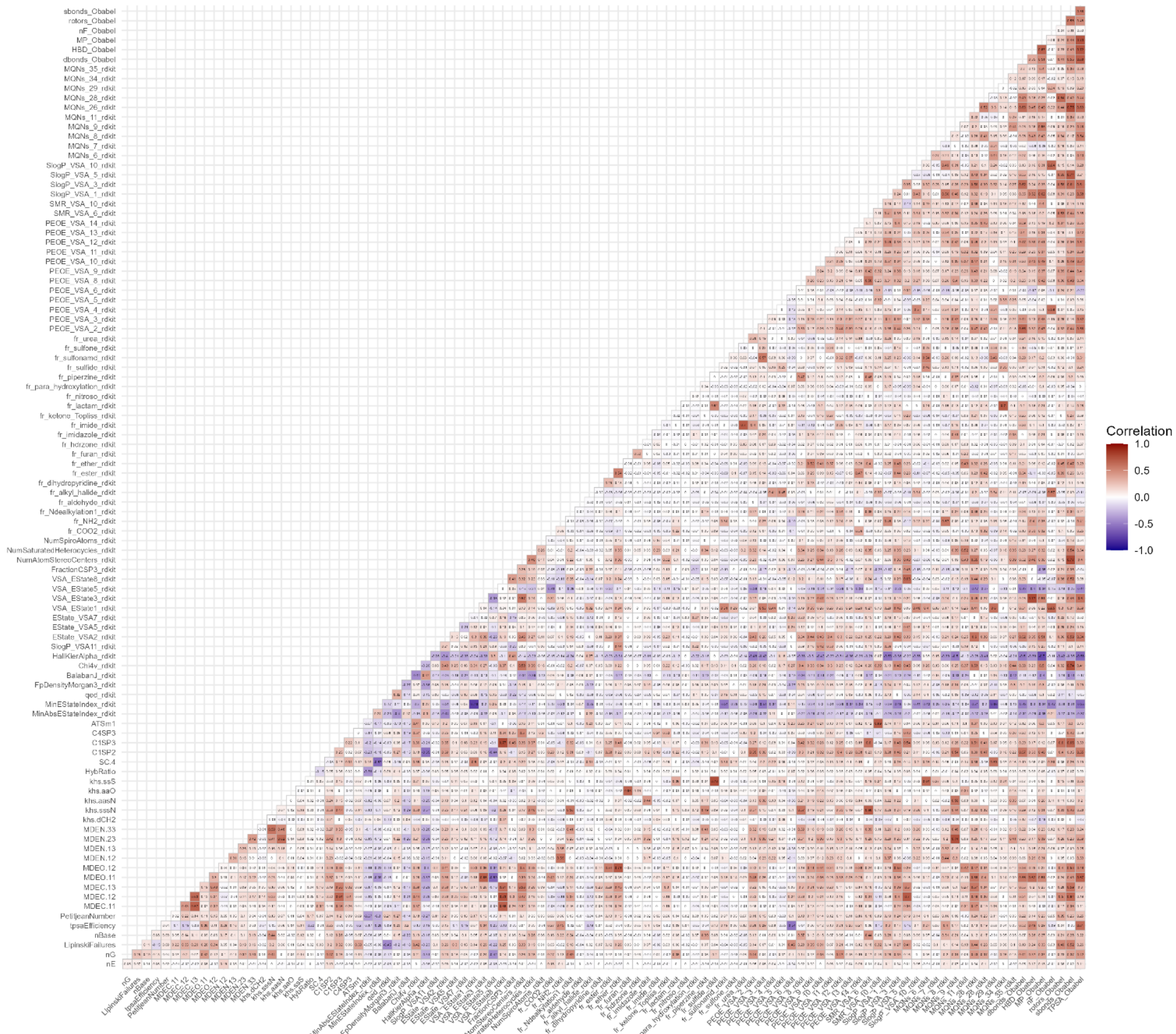
Correlation
1.0
0.5
0.0
-0.5
-1.0

Figure S2. Correlation coefficients between the selected descriptors after the Univariate Feature Selection.

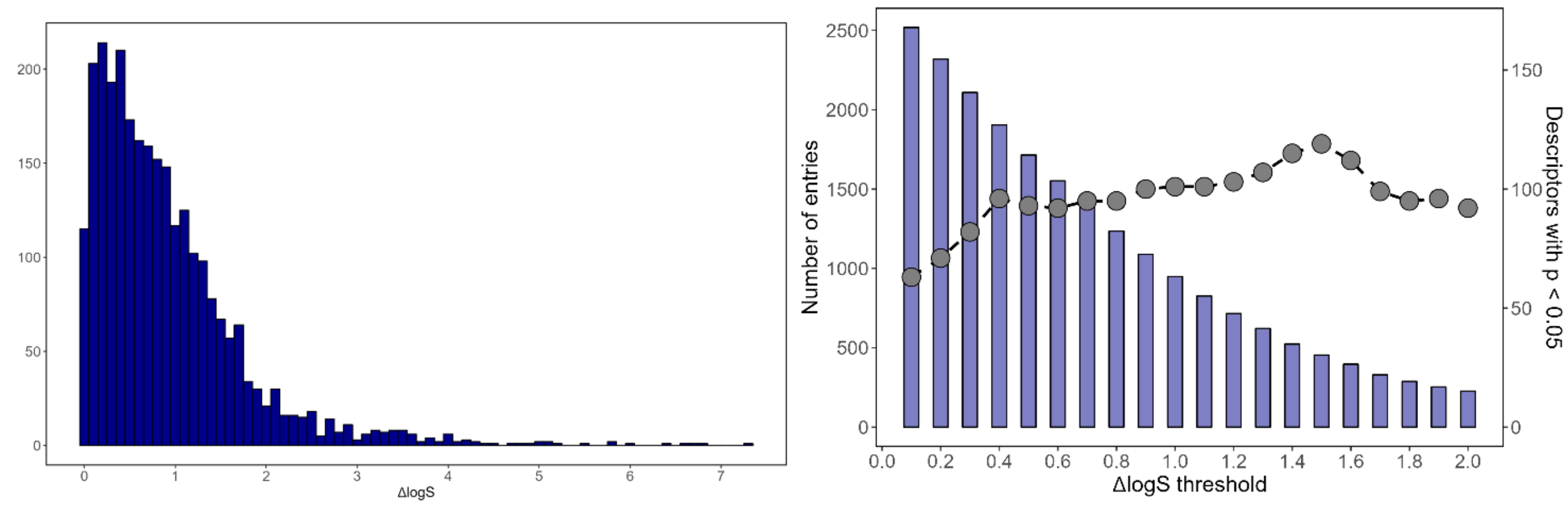

Figure S3. (*left*) Distribution of $\Delta \log S$ values after evaluating the GSE in SolCbio3Database. (*right*) **Columns**: Amount of data entries that indicate a GSE prediction where $\Delta \log S \leq i$ (left vertical axis). **Points**: Number of descriptors for each tested $\Delta \log S$ threshold that showed significant separation between groups through the UFS.

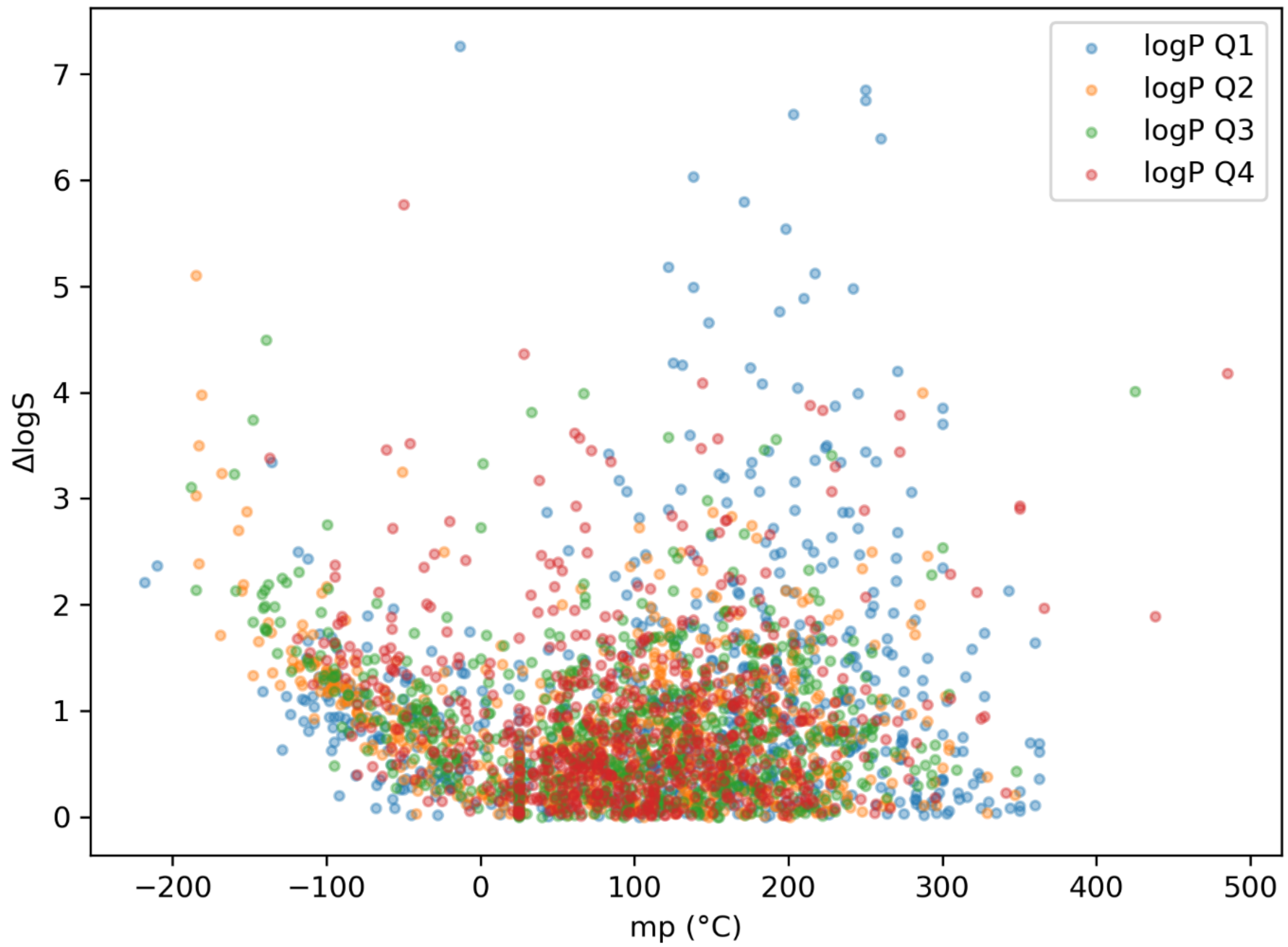

Figure S4. Distribution of $\log P_N$ and *mp* values in function of $\Delta \log S$, from comparing experimental and GSE-estimated intrinsic solubility values in SolCbio3Database.

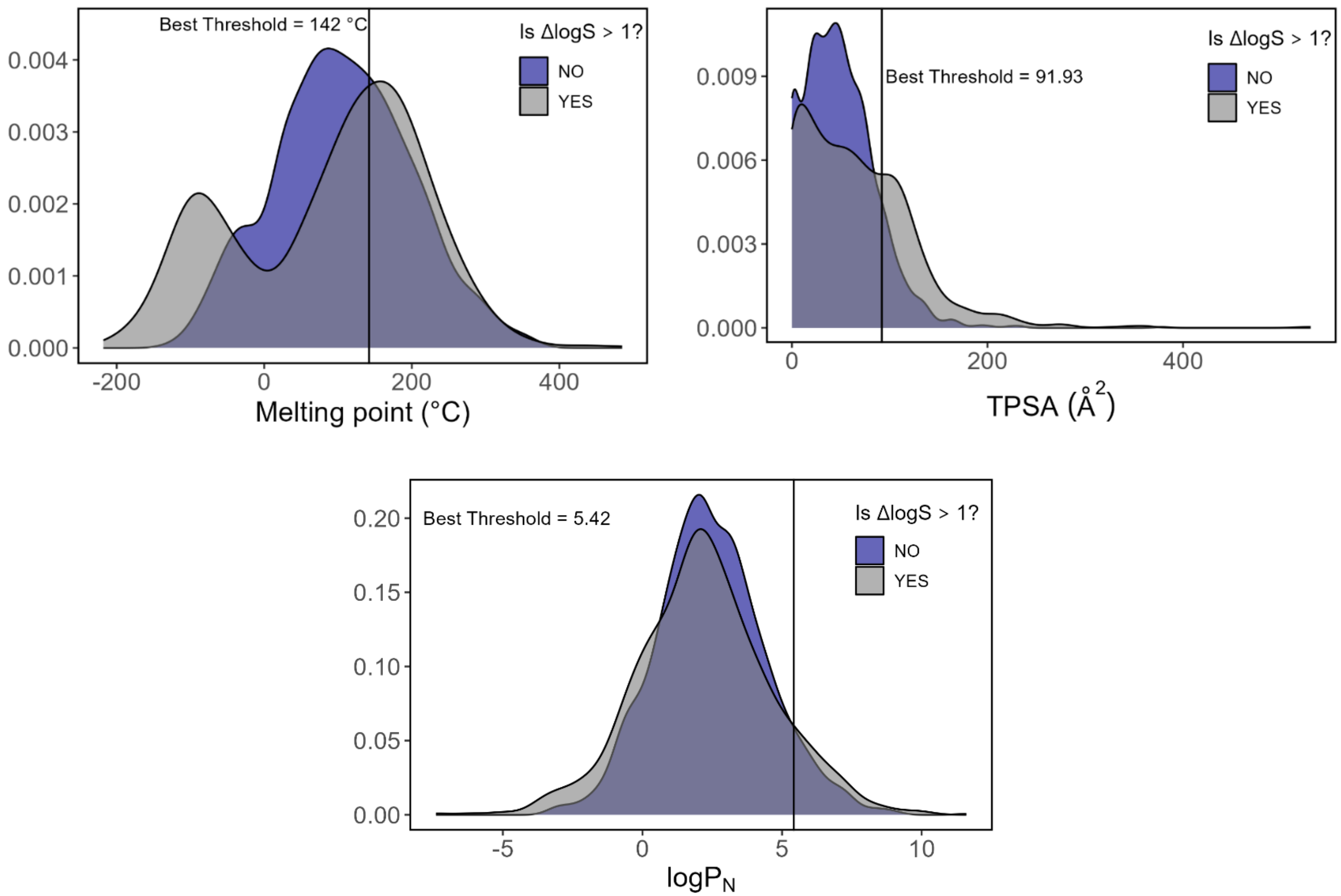

Figure S5. Density plots that integrate the optimal decision thresholds for an empirical rule-based model for the GSE viability based on the melting point (*top left*), TPSA (*top right*), and log P_N (*bottom*) of SolCbio3Database after an iterative Youden's J statistic algorithm (optimal values: *mp* = 142 °C, TPSA = 91.93 Å^2, log P_N = 5.42).

Table S1. Selected descriptors after the Univariate Feature Selection performed by performing a two tailed Welch's *t*-test. Documentation for each descriptor is provided.

Descriptor	p-value (Welch's *t*-test)	Source	Reference link
nE	0.045443	RCDK	https://cdk.github.io/cdk/2.3/docs/api/org/openscience/cdk/qsar/descriptors/molecular/AminoAcidCountDescriptor.html
nG	8.96E-05	RCDK	https://cdk.github.io/cdk/2.3/docs/api/org/openscience/cdk/qsar/descriptors/molecular/AminoAcidCountDescriptor.html
LipinskiFailures	6.48E-10	RCDK	https://cdk.github.io/cdk/2.3/docs/api/org/openscience/cdk/qsar/descriptors/molecular/RuleOfFiveDescriptor.html
nBase	0.002247	RCDK	https://cdk.github.io/cdk/2.3/docs/api/org/openscience/cdk/qsar/descriptors/molecular/BasicGroupCountDescriptor.html
tpsaEfficiency	0.0283	RCDK	https://cdk.github.io/cdk/1.5/docs/api/org/openscience/cdk/qsar/descriptors/molecular/FractionalPSADescriptor.html
PetitjeanNumber	0.009967	RCDK	https://cdk.github.io/cdk/2.3/docs/api/org/openscience/cdk/qsar/descriptors/molecular/PetitjeanNumberDescriptor.html
MDEC.11	0.019687	RCDK	https://cdk.github.io/cdk/2.3/docs/api/org/openscience/cdk/qsar/descriptors/molecular/MDEDescriptor.html
MDEC.12	0.000774	RCDK	https://cdk.github.io/cdk/2.3/docs/api/org/openscience/cdk/qsar/descriptors/molecular/MDEDescriptor.html
MDEC.13	0.015963	RCDK	https://cdk.github.io/cdk/2.3/docs/api/org/openscience/cdk/qsar/descriptors/molecular/MDEDescriptor.html
MDEO.11	9.47E-09	RCDK	https://cdk.github.io/cdk/2.3/docs/api/org/openscience/cdk/qsar/descriptors/molecular/MDEDescriptor.html
MDEO.12	1.37E-09	RCDK	https://cdk.github.io/cdk/2.3/docs/api/org/openscience/cdk/qsar/descriptors/molecular/MDEDescriptor.html
MDEN.12	0.039091	RCDK	https://cdk.github.io/cdk/2.3/docs/api/org/openscience/cdk/qsar/descriptors/molecular/MDEDescriptor.html
MDEN.13	0.025821	RCDK	https://cdk.github.io/cdk/2.3/docs/api/org/openscience/cdk/qsar/descriptors/molecular/MDEDescriptor.html
MDEN.23	9.98E-07	RCDK	https://cdk.github.io/cdk/2.3/docs/api/org/openscience/cdk/qsar/descriptors/molecular/MDEDescriptor.html
MDEN.33	0.001483	RCDK	https://cdk.github.io/cdk/2.3/docs/api/org/openscience/cdk/qsar/descriptors/molecular/MDEDescriptor.html
khs.dCH2	0.000809	RCDK	https://cdk.github.io/cdk/2.3/docs/api/org/openscience/cdk/qsar/descriptors/molecular/KierHallSmartsDescriptor.html
khs.sssN	0.000576	RCDK	https://cdk.github.io/cdk/2.3/docs/api/org/openscience/cdk/qsar/descriptors/molecular/KierHallSmartsDescriptor.html
khs.aasN	0.002298	RCDK	https://cdk.github.io/cdk/2.3/docs/api/org/openscience/cdk/qsar/descriptors/molecular/KierHallSmartsDescriptor.html
khs.aaO	0.01163	RCDK	https://cdk.github.io/cdk/2.3/docs/api/org/openscience/cdk/qsar/descriptors/molecular/KierHallSmartsDescriptor.html

Descriptor	p-value (Welch's *t*-test)	Source	Reference link
khs.ssS	0.000545	RCDK	https://cdk.github.io/cdk/2.3/docs/api/org/openscience/cdk/qsar/descriptors/molecular/KierHallSmartsDescriptor.html
HybRatio	0.030135	RCDK	https://cdk.github.io/cdk/2.3/docs/api/org/openscience/cdk/qsar/descriptors/molecular/HybridizationRatioDescriptor.html
SC.4	0.000585	RCDK	https://cdk.github.io/cdk/2.3/docs/api/org/openscience/cdk/qsar/descriptors/molecular/ChiClusterDescriptor.html
C1SP2	7.71E-11	RCDK	https://cdk.github.io/cdk/2.3/docs/api/org/openscience/cdk/qsar/descriptors/molecular/CarbonTypesDescriptor.html
C1SP3	2.90E-09	RCDK	https://cdk.github.io/cdk/2.3/docs/api/org/openscience/cdk/qsar/descriptors/molecular/CarbonTypesDescriptor.html
C4SP3	0.00596	RCDK	https://cdk.github.io/cdk/2.3/docs/api/org/openscience/cdk/qsar/descriptors/molecular/CarbonTypesDescriptor.html
ATSm1	1.06E-05	RCDK	https://cdk.github.io/cdk/2.3/docs/api/org/openscience/cdk/qsar/descriptors/molecular/AutocorrelationDescriptorMass.html
MinAbsEStateIndex_rdkit	0.032602	RDKit	https://www.rdkit.org/docs/source/rdkit.Chem.EState.EState.html#rdkit.Chem.EState.EState
MinEStateIndex_rdkit	1.11E-08	RDKit	https://www.rdkit.org/docs/source/rdkit.Chem.EState.EState.html#rdkit.Chem.EState.EState
qed_rdkit	2.39E-19	RDKit	https://www.rdkit.org/docs/source/rdkit.Chem.QED.html#rdkit.Chem.QED.qed
FpDensityMorgan3_rdkit	0.013579	RDKit	https://www.rdkit.org/docs/source/rdkit.Chem.Descriptors.html#rdkit.Chem.Descriptors.FpDensityMorgan3
BalabanJ_rdkit	0.00056	RDKit	https://www.rdkit.org/docs/source/rdkit.Chem.GraphDescriptors.html#rdkit.Chem
Chi4v_rdkit	0.000165	RDKit	https://www.rdkit.org/docs/source/rdkit.Chem.GraphDescriptors.html#rdkit.Chem
HallKierAlpha_rdkit	0.02419	RDKit	https://www.rdkit.org/docs/source/rdkit.Chem.GraphDescriptors.html#rdkit.Chem , also check 10.1002/med.2610070404 [1]
SlogP_VSA11_rdkit	0.002797	RDKit	https://www.rdkit.org/docs/source/rdkit.Chem.MolSurf.html#rdkit.Chem.MolSurf , also check 10.1021/ci990307l [2]
EState_VSA2_rdkit	1.63E-10	RDKit	https://www.rdkit.org/docs/source/rdkit.Chem.EState.EState_VSA.html#rdkit.Chem.EState , also check 10.1021/ci00028a014 [3]
EState_VSA5_rdkit	8.65E-09	RDKit	https://www.rdkit.org/docs/source/rdkit.Chem.EState.EState_VSA.html#rdkit.Chem.EState , also check 10.1021/ci00028a014 [3]
EState_VSA7_rdkit	0.000365	RDKit	https://www.rdkit.org/docs/source/rdkit.Chem.EState.EState_VSA.html#rdkit.Chem.EState , also check 10.1021/ci00028a014 [3]
VSA_EState1_rdkit	1.77E-15	RDKit	https://www.rdkit.org/docs/source/rdkit.Chem.EState.EState_VSA.html#rdkit.Chem.EState , also check 10.1021/ci00028a014 [3]
VSA_EState3_rdkit	0.001026	RDKit	https://www.rdkit.org/docs/source/rdkit.Chem.EState.EState_VSA.html#rdkit.Chem.EState , also check 10.1021/ci00028a014 [3]
VSA_EState5_rdkit	8.08E-05	RDKit	https://www.rdkit.org/docs/source/rdkit.Chem.EState.EState_VSA.html#rdkit.Chem.EState , also check 10.1021/ci00028a014 [3]
VSA_EState8_rdkit	0.002256	RDKit	https://www.rdkit.org/docs/source/rdkit.Chem.EState.EState_VSA.html#rdkit.Chem.EState , also check 10.1021/ci00028a014 [3]
FractionCSP3_rdkit	2.80E-07	RDKit	https://www.rdkit.org/docs/source/rdkit.Chem.Lipinski.html#rdkit.Chem.Lipinski
NumAtomStereoCenters_rdkit	0.021848	RDKit	https://www.rdkit.org/docs/source/rdkit.Chem.Lipinski.html#rdkit.Chem.Lipinski

**Descriptor**	**p-value (Welch's *t*-test)**	**Source**	**Reference link**
NumSaturatedHeterocycles_rdkit	0.007743	RDKit	https://www.rdkit.org/docs/source/rdkit.Chem.Lipinski.html#rdkit.Chem.Lipinski
NumSpiroAtoms_rdkit	0.044958	RDKit	https://www.rdkit.org/docs/source/rdkit.Chem.Lipinski.html#rdkit.Chem.Lipinski
fr_COO2_rdkit	0.028745	RDKit	https://www.rdkit.org/docs/source/rdkit.Chem.Fragments.html#rdkit.Chem.Fragments
fr_NH2_rdkit	0.028995	RDKit	https://www.rdkit.org/docs/source/rdkit.Chem.Fragments.html#rdkit.Chem.Fragments
fr_Ndealkylation1_rdkit	0.009379	RDKit	https://www.rdkit.org/docs/source/rdkit.Chem.Fragments.html#rdkit.Chem.Fragments
fr_aldehyde_rdkit	0.037936	RDKit	https://www.rdkit.org/docs/source/rdkit.Chem.Fragments.html#rdkit.Chem.Fragments
fr_alkyl_halide_rdkit	0.00658	RDKit	https://www.rdkit.org/docs/source/rdkit.Chem.Fragments.html#rdkit.Chem.Fragments
fr_dihydropyridine_rdkit	0.045286	RDKit	https://www.rdkit.org/docs/source/rdkit.Chem.Fragments.html#rdkit.Chem.Fragments
fr_ester_rdkit	2.95E-05	RDKit	https://www.rdkit.org/docs/source/rdkit.Chem.Fragments.html#rdkit.Chem.Fragments
fr_ether_rdkit	2.28E-08	RDKit	https://www.rdkit.org/docs/source/rdkit.Chem.Fragments.html#rdkit.Chem.Fragments
fr_furan_rdkit	0.031714	RDKit	https://www.rdkit.org/docs/source/rdkit.Chem.Fragments.html#rdkit.Chem.Fragments
fr_hdrzone_rdkit	0.030972	RDKit	https://www.rdkit.org/docs/source/rdkit.Chem.Fragments.html#rdkit.Chem.Fragments
fr_imidazole_rdkit	0.002193	RDKit	https://www.rdkit.org/docs/source/rdkit.Chem.Fragments.html#rdkit.Chem.Fragments
fr_imide_rdkit	0.000461	RDKit	https://www.rdkit.org/docs/source/rdkit.Chem.Fragments.html#rdkit.Chem.Fragments
fr_ketone_Topliss_rdkit	0.024671	RDKit	https://www.rdkit.org/docs/source/rdkit.Chem.Fragments.html#rdkit.Chem.Fragments
fr_lactam_rdkit	0.014955	RDKit	https://www.rdkit.org/docs/source/rdkit.Chem.Fragments.html#rdkit.Chem.Fragments
fr_nitroso_rdkit	0.001549	RDKit	https://www.rdkit.org/docs/source/rdkit.Chem.Fragments.html#rdkit.Chem.Fragments
fr_para_hydroxylation_rdkit	0.01153	RDKit	https://www.rdkit.org/docs/source/rdkit.Chem.Fragments.html#rdkit.Chem.Fragments
fr_piperzine_rdkit	0.007045	RDKit	https://www.rdkit.org/docs/source/rdkit.Chem.Fragments.html#rdkit.Chem.Fragments
fr_sulfide_rdkit	0.000663	RDKit	https://www.rdkit.org/docs/source/rdkit.Chem.Fragments.html#rdkit.Chem.Fragments
fr_sulfonamd_rdkit	0.001231	RDKit	https://www.rdkit.org/docs/source/rdkit.Chem.Fragments.html#rdkit.Chem.Fragments
fr_sulfone_rdkit	0.00567	RDKit	https://www.rdkit.org/docs/source/rdkit.Chem.Fragments.html#rdkit.Chem.Fragments
fr_urea_rdkit	0.022241	RDKit	https://www.rdkit.org/docs/source/rdkit.Chem.Fragments.html#rdkit.Chem.Fragments
PEOE_VSA_2_rdkit	0.025938	RDKit	https://www.rdkit.org/docs/source/rdkit.Chem.MolSurf.html, also check 10.1016/0040-4020(80)80168-2 [4]

Descriptor	p-value (Welch's *t*-test)	Source	Reference link
PEOE_VSA_3_rdkit	1.61E-11	RDKit	https://www.rdkit.org/docs/source/rdkit.Chem.MolSurf.html, also check 10.1016/0040-4020(80)80168-2 [4]
PEOE_VSA_4_rdkit	0.000433	RDKit	https://www.rdkit.org/docs/source/rdkit.Chem.MolSurf.html, also check 10.1016/0040-4020(80)80168-2 [4]
PEOE_VSA_5_rdkit	0.000153	RDKit	https://www.rdkit.org/docs/source/rdkit.Chem.MolSurf.html, also check 10.1016/0040-4020(80)80168-2 [4]
PEOE_VSA_6_rdkit	0.030583	RDKit	https://www.rdkit.org/docs/source/rdkit.Chem.MolSurf.html, also check 10.1016/0040-4020(80)80168-2 [4]
PEOE_VSA_8_rdkit	0.017786	RDKit	https://www.rdkit.org/docs/source/rdkit.Chem.MolSurf.html, also check 10.1016/0040-4020(80)80168-2 [4]
PEOE_VSA_9_rdkit	5.60E-11	RDKit	https://www.rdkit.org/docs/source/rdkit.Chem.MolSurf.html, also check 10.1016/0040-4020(80)80168-2 [4]
PEOE_VSA_10_rdkit	2.44E-11	RDKit	https://www.rdkit.org/docs/source/rdkit.Chem.MolSurf.html, also check 10.1016/0040-4020(80)80168-2 [4]
PEOE_VSA_11_rdkit	0.001567	RDKit	https://www.rdkit.org/docs/source/rdkit.Chem.MolSurf.html, also check 10.1016/0040-4020(80)80168-2 [4]
PEOE_VSA_12_rdkit	0.001015	RDKit	https://www.rdkit.org/docs/source/rdkit.Chem.MolSurf.html, also check 10.1016/0040-4020(80)80168-2 [4]
PEOE_VSA_13_rdkit	0.00028	RDKit	https://www.rdkit.org/docs/source/rdkit.Chem.MolSurf.html, also check 10.1016/0040-4020(80)80168-2 [4]
PEOE_VSA_14_rdkit	5.48E-07	RDKit	https://www.rdkit.org/docs/source/rdkit.Chem.MolSurf.html, also check 10.1016/0040-4020(80)80168-2 [4]
SMR_VSA_6_rdkit	5.46E-10	RDKit	https://www.rdkit.org/docs/source/rdkit.Chem.MolSurf.html, also check 10.1021/ci990307l [2]
SMR_VSA_10_rdkit	4.47E-05	RDKit	https://www.rdkit.org/docs/source/rdkit.Chem.MolSurf.html, also check 10.1021/ci990307l [2]
SlogP_VSA_1_rdkit	0.000251	RDKit	https://www.rdkit.org/docs/source/rdkit.Chem.MolSurf.html, also check 10.1021/ci990307l [2]
SlogP_VSA_3_rdkit	1.64E-12	RDKit	https://www.rdkit.org/docs/source/rdkit.Chem.MolSurf.html, also check 10.1021/ci990307l [2]
SlogP_VSA_5_rdkit	0.005648	RDKit	https://www.rdkit.org/docs/source/rdkit.Chem.MolSurf.html, also check 10.1021/ci990307l [2]
SlogP_VSA_10_rdkit	0.02714	RDKit	https://www.rdkit.org/docs/source/rdkit.Chem.MolSurf.html, also check 10.1021/ci990307l [2]
MQNs_6_rdkit	1.59E-08	RDKit	https://www.rdkit.org/docs/source/rdkit.Chem.rdMolDescriptors.html#rdkit.Chem.rdMolDescriptors.MQNs_, also check 10.1002/cmdc.200900317 [5]
MQNs_7_rdkit	0.005868	RDKit	https://www.rdkit.org/docs/source/rdkit.Chem.rdMolDescriptors.html#rdkit.Chem.rdMolDescriptors.MQNs_, also check 10.1002/cmdc.200900317 [5]
MQNs_8_rdkit	7.34E-05	RDKit	https://www.rdkit.org/docs/source/rdkit.Chem.rdMolDescriptors.html#rdkit.Chem.rdMolDescriptors.MQNs_, also check 10.1002/cmdc.200900317 [5]
MQNs_9_rdkit	0.000324	RDKit	https://www.rdkit.org/docs/source/rdkit.Chem.rdMolDescriptors.html#rdkit.Chem.rdMolDescriptors.MQNs_, also check 10.1002/cmdc.200900317 [5]
MQNs_11_rdkit	0.017541	RDKit	https://www.rdkit.org/docs/source/rdkit.Chem.rdMolDescriptors.html#rdkit.Chem.rdMolDescriptors.MQNs_, also check 10.1002/cmdc.200900317 [5]
MQNs_26_rdkit	6.57E-14	RDKit	https://www.rdkit.org/docs/source/rdkit.Chem.rdMolDescriptors.html#rdkit.Chem.rdMolDescriptors.MQNs_, also check 10.1002/cmdc.200900317 [5]
MQNs_28_rdkit	5.14E-07	RDKit	https://www.rdkit.org/docs/source/rdkit.Chem.rdMolDescriptors.html#rdkit.Chem.rdMolDescriptors.MQNs_, also check 10.1002/cmdc.200900317 [5]

Descriptor	p-value (Welch's *t*-test)	Source	Reference link
MQNs_29_rdkit	3.84E-07	RDKit	https://www.rdkit.org/docs/source/rdkit.Chem.rdMolDescriptors.html#rdkit.Chem.rdMolDescriptors.MQNs_, also check 10.1002/cmdc.200900317 [5]
MQNs_34_rdkit	0.008294	RDKit	https://www.rdkit.org/docs/source/rdkit.Chem.rdMolDescriptors.html#rdkit.Chem.rdMolDescriptors.MQNs_, also check 10.1002/cmdc.200900317 [5]
MQNs_35_rdkit	0.002784	RDKit	https://www.rdkit.org/docs/source/rdkit.Chem.rdMolDescriptors.html#rdkit.Chem.rdMolDescriptors.MQNs_, also check 10.1002/cmdc.200900317 [5]
dbonds_Obabel	3.68E-09	OpenBabel	https://open-babel.readthedocs.io/en/latest/Descriptors/descriptors.html
HBD_Obabel	0.001688	OpenBabel	https://open-babel.readthedocs.io/en/latest/Descriptors/descriptors.html
MP_Obabel	0.00067	OpenBabel	https://open-babel.readthedocs.io/en/latest/Descriptors/descriptors.html
nF_Obabel	0.003001	OpenBabel	https://open-babel.readthedocs.io/en/latest/Descriptors/descriptors.html
rotors_Obabel	5.21E-16	OpenBabel	https://open-babel.readthedocs.io/en/latest/Descriptors/descriptors.html
sbonds_Obabel	1.10E-09	OpenBabel	https://open-babel.readthedocs.io/en/latest/Descriptors/descriptors.html
TPSA_Obabel	2.41E-15	OpenBabel	https://open-babel.readthedocs.io/en/latest/Descriptors/descriptors.html

Table S2. Selected descriptors to train the classification ML models after the Recursive Feature Elimination.

Descriptor	Source	Reference link
nBase	RCDK	https://cdk.github.io/cdk/2.3/docs/api/org/openscience/cdk/qsar/descriptors/molecular/BasicGroupCountDescriptor.html
tpsaEfficiency	RCDK	https://cdk.github.io/cdk/1.5/docs/api/org/openscience/cdk/qsar/descriptors/molecular/FractionalPSADescriptor.html
PetitjeanNumber	RCDK	https://cdk.github.io/cdk/2.3/docs/api/org/openscience/cdk/qsar/descriptors/molecular/PetitjeanNumberDescriptor.html
MDEC.11	RCDK	https://cdk.github.io/cdk/2.3/docs/api/org/openscience/cdk/qsar/descriptors/molecular/MDEDescriptor.html
MDEC.12	RCDK	https://cdk.github.io/cdk/2.3/docs/api/org/openscience/cdk/qsar/descriptors/molecular/MDEDescriptor.html
MDEC.13	RCDK	https://cdk.github.io/cdk/2.3/docs/api/org/openscience/cdk/qsar/descriptors/molecular/MDEDescriptor.html
MDEO.11	RCDK	https://cdk.github.io/cdk/2.3/docs/api/org/openscience/cdk/qsar/descriptors/molecular/MDEDescriptor.html
MDEO.12	RCDK	https://cdk.github.io/cdk/2.3/docs/api/org/openscience/cdk/qsar/descriptors/molecular/MDEDescriptor.html
MDEN.23	RCDK	https://cdk.github.io/cdk/2.3/docs/api/org/openscience/cdk/qsar/descriptors/molecular/MDEDescriptor.html
HybRatio	RCDK	https://cdk.github.io/cdk/2.3/docs/api/org/openscience/cdk/qsar/descriptors/molecular/KierHallSmartsDescriptor.html
SC.4	RCDK	https://cdk.github.io/cdk/2.3/docs/api/org/openscience/cdk/qsar/descriptors/molecular/ChiClusterDescriptor.html
C1SP2	RCDK	https://cdk.github.io/cdk/2.3/docs/api/org/openscience/cdk/qsar/descriptors/molecular/CarbonTypesDescriptor.html
C1SP3	RCDK	https://cdk.github.io/cdk/2.3/docs/api/org/openscience/cdk/qsar/descriptors/molecular/CarbonTypesDescriptor.html
ATSm1	RCDK	https://cdk.github.io/cdk/2.3/docs/api/org/openscience/cdk/qsar/descriptors/molecular/AutocorrelationDescriptorMass.html
MinAbsEStateIndex_rdkit	RDKit	https://www.rdkit.org/docs/source/rdkit.Chem.EState.EState.html#rdkit.Chem.EState.EState
MinEStateIndex_rdkit	RDKit	https://www.rdkit.org/docs/source/rdkit.Chem.EState.EState.html#rdkit.Chem.EState.EState
qed_rdkit	RDKit	https://www.rdkit.org/docs/source/rdkit.Chem.QED.html#rdkit.Chem.QED.qed
FpDensityMorgan3_rdkit	RDKit	https://www.rdkit.org/docs/source/rdkit.Chem.Descriptors.html#rdkit.Chem.Descriptors.FpDensityMorgan3
BalabanJ_rdkit	RDKit	https://www.rdkit.org/docs/source/rdkit.Chem.GraphDescriptors.html#rdkit.Chem
Chi4v_rdkit	RDKit	https://www.rdkit.org/docs/source/rdkit.Chem.GraphDescriptors.html#rdkit.Chem
HallKierAlpha_rdkit	RDKit	https://www.rdkit.org/docs/source/rdkit.Chem.GraphDescriptors.html#rdkit.Chem

Descriptor	Source	Reference link
SlogP_VSA11_rdkit	RDKit	https://www.rdkit.org/docs/source/rdkit.Chem.MolSurf.html#rdkit.Chem.MolSurf , also check 10.1021/ci990307l [2]
EState_VSA2_rdkit	RDKit	https://www.rdkit.org/docs/source/rdkit.Chem.EState.EState_VSA.html#rdkit.Chem.EState , also check 10.1021/ci00028a014 [3]
EState_VSA5_rdkit	RDKit	https://www.rdkit.org/docs/source/rdkit.Chem.EState.EState_VSA.html#rdkit.Chem.EState , also check 10.1021/ci00028a014 [3]
EState_VSA7_rdkit	RDKit	https://www.rdkit.org/docs/source/rdkit.Chem.EState.EState_VSA.html#rdkit.Chem.EState , also check 10.1021/ci00028a014 [3]
VSA_EState1_rdkit	RDKit	https://www.rdkit.org/docs/source/rdkit.Chem.EState.EState_VSA.html#rdkit.Chem.EState , also check 10.1021/ci00028a014 [3]
VSA_EState3_rdkit	RDKit	https://www.rdkit.org/docs/source/rdkit.Chem.EState.EState_VSA.html#rdkit.Chem.EState , also check 10.1021/ci00028a014 [3]
VSA_EState5_rdkit	RDKit	https://www.rdkit.org/docs/source/rdkit.Chem.EState.EState_VSA.html#rdkit.Chem.EState , also check 10.1021/ci00028a014 [3]
VSA_EState8_rdkit	RDKit	https://www.rdkit.org/docs/source/rdkit.Chem.EState.EState_VSA.html#rdkit.Chem.EState , also check 10.1021/ci00028a014 [3]
FractionCSP3_rdkit	RDKit	https://www.rdkit.org/docs/source/rdkit.Chem.Lipinski.html#rdkit.Chem.Lipinski
NumAtomStereoCenters_rdkit	RDKit	https://www.rdkit.org/docs/source/rdkit.Chem.Lipinski.html#rdkit.Chem.Lipinski
fr_ether_rdkit	RDKit	https://www.rdkit.org/docs/source/rdkit.Chem.Fragments.html#rdkit.Chem.Fragments
PEOE_VSA_2_rdkit	RDKit	https://www.rdkit.org/docs/source/rdkit.Chem.MolSurf.html, also check 10.1016/0040-4020(80)80168-2 [4]
PEOE_VSA_3_rdkit	RDKit	https://www.rdkit.org/docs/source/rdkit.Chem.MolSurf.html, also check 10.1016/0040-4020(80)80168-2 [4]
PEOE_VSA_4_rdkit	RDKit	https://www.rdkit.org/docs/source/rdkit.Chem.MolSurf.html, also check 10.1016/0040-4020(80)80168-2 [4]
PEOE_VSA_5_rdkit	RDKit	https://www.rdkit.org/docs/source/rdkit.Chem.MolSurf.html, also check 10.1016/0040-4020(80)80168-2 [4]
PEOE_VSA_6_rdkit	RDKit	https://www.rdkit.org/docs/source/rdkit.Chem.MolSurf.html, also check 10.1016/0040-4020(80)80168-2 [4]
PEOE_VSA_8_rdkit	RDKit	https://www.rdkit.org/docs/source/rdkit.Chem.MolSurf.html, also check 10.1016/0040-4020(80)80168-2 [4]
PEOE_VSA_9_rdkit	RDKit	https://www.rdkit.org/docs/source/rdkit.Chem.MolSurf.html, also check 10.1016/0040-4020(80)80168-2 [4]
PEOE_VSA_10_rdkit	RDKit	https://www.rdkit.org/docs/source/rdkit.Chem.MolSurf.html, also check 10.1016/0040-4020(80)80168-2 [4]
PEOE_VSA_11_rdkit	RDKit	https://www.rdkit.org/docs/source/rdkit.Chem.MolSurf.html, also check 10.1016/0040-4020(80)80168-2 [4]
PEOE_VSA_12_rdkit	RDKit	https://www.rdkit.org/docs/source/rdkit.Chem.MolSurf.html, also check 10.1016/0040-4020(80)80168-2 [4]
PEOE_VSA_13_rdkit	RDKit	https://www.rdkit.org/docs/source/rdkit.Chem.MolSurf.html, also check 10.1016/0040-4020(80)80168-2 [4]

Descriptor	Source	Reference link
PEOE_VSA_14_rdkit	RDKit	https://www.rdkit.org/docs/source/rdkit.Chem.MolSurf.html, also check 10.1016/0040-4020(80)80168-2 [4]
SMR_VSA_6_rdkit	RDKit	https://www.rdkit.org/docs/source/rdkit.Chem.MolSurf.html, also check 10.1021/ci990307l [2]
SMR_VSA_10_rdkit	RDKit	https://www.rdkit.org/docs/source/rdkit.Chem.MolSurf.html, also check 10.1021/ci990307l [2]
SlogP_VSA_1_rdkit	RDKit	https://www.rdkit.org/docs/source/rdkit.Chem.MolSurf.html, also check 10.1021/ci990307l [2]
SlogP_VSA_3_rdkit	RDKit	https://www.rdkit.org/docs/source/rdkit.Chem.MolSurf.html, also check 10.1021/ci990307l [2]
SlogP_VSA_5_rdkit	RDKit	https://www.rdkit.org/docs/source/rdkit.Chem.MolSurf.html, also check 10.1021/ci990307l [2]
SlogP_VSA_10_rdkit	RDKit	https://www.rdkit.org/docs/source/rdkit.Chem.MolSurf.html, also check 10.1021/ci990307l [2]
MQNs_6_rdkit	RDKit	https://www.rdkit.org/docs/source/rdkit.Chem.rdMolDescriptors.html#rdkit.Chem.rdMolDescriptors.MQNs_, also check 10.1002/cmdc.200900317 [5]
MQNs_8_rdkit	RDKit	https://www.rdkit.org/docs/source/rdkit.Chem.rdMolDescriptors.html#rdkit.Chem.rdMolDescriptors.MQNs_, also check 10.1002/cmdc.200900317 [5]
MQNs_9_rdkit	RDKit	https://www.rdkit.org/docs/source/rdkit.Chem.rdMolDescriptors.html#rdkit.Chem.rdMolDescriptors.MQNs_, also check 10.1002/cmdc.200900317 [5]
MQNs_26_rdkit	RDKit	https://www.rdkit.org/docs/source/rdkit.Chem.rdMolDescriptors.html#rdkit.Chem.rdMolDescriptors.MQNs_, also check 10.1002/cmdc.200900317 [5]
MQNs_28_rdkit	RDKit	https://www.rdkit.org/docs/source/rdkit.Chem.rdMolDescriptors.html#rdkit.Chem.rdMolDescriptors.MQNs_, also check 10.1002/cmdc.200900317 [5]
MQNs_35_rdkit	RDKit	https://www.rdkit.org/docs/source/rdkit.Chem.rdMolDescriptors.html#rdkit.Chem.rdMolDescriptors.MQNs_, also check 10.1002/cmdc.200900317 [5]
dbonds_Obabel	OpenBabel	https://open-babel.readthedocs.io/en/latest/Descriptors/descriptors.html
HBD_Obabel	OpenBabel	https://open-babel.readthedocs.io/en/latest/Descriptors/descriptors.html
MP_Obabel	OpenBabel	https://open-babel.readthedocs.io/en/latest/Descriptors/descriptors.html
rotors_Obabel	OpenBabel	https://open-babel.readthedocs.io/en/latest/Descriptors/descriptors.html
sbonds_Obabel	OpenBabel	https://open-babel.readthedocs.io/en/latest/Descriptors/descriptors.html
TPSA_Obabel	OpenBabel	https://open-babel.readthedocs.io/en/latest/Descriptors/descriptors.html
morgan_fp_32	Morgan Fingerprint	https://www.rdkit.org/docs/source/rdkit.Chem.AllChem.html, also check our morgan_fingerprint_generator.py file in our repository.
morgan_fp_64	Morgan Fingerprint	https://www.rdkit.org/docs/source/rdkit.Chem.AllChem.html, also check our morgan_fingerprint_generator.py file in our repository.
morgan_fp_79	Morgan Fingerprint	https://www.rdkit.org/docs/source/rdkit.Chem.AllChem.html, also check our morgan_fingerprint_generator.py file in our repository.

Descriptor	Source	Reference link
morgan_fp_174	Morgan Fingerprint	https://www.rdkit.org/docs/source/rdkit.Chem.AllChem.html, also check our morgan_fingerprint_generator.py file in our repository.
morgan_fp_355	Morgan Fingerprint	https://www.rdkit.org/docs/source/rdkit.Chem.AllChem.html, also check our morgan_fingerprint_generator.py file in our repository.
morgan_fp_577	Morgan Fingerprint	https://www.rdkit.org/docs/source/rdkit.Chem.AllChem.html, also check our morgan_fingerprint_generator.py file in our repository.
morgan_fp_649	Morgan Fingerprint	https://www.rdkit.org/docs/source/rdkit.Chem.AllChem.html, also check our morgan_fingerprint_generator.py file in our repository.
morgan_fp_694	Morgan Fingerprint	https://www.rdkit.org/docs/source/rdkit.Chem.AllChem.html, also check our morgan_fingerprint_generator.py file in our repository.
morgan_fp_725	Morgan Fingerprint	https://www.rdkit.org/docs/source/rdkit.Chem.AllChem.html, also check our morgan_fingerprint_generator.py file in our repository.
morgan_fp_744	Morgan Fingerprint	https://www.rdkit.org/docs/source/rdkit.Chem.AllChem.html, also check our morgan_fingerprint_generator.py file in our repository.
morgan_fp_806	Morgan Fingerprint	https://www.rdkit.org/docs/source/rdkit.Chem.AllChem.html, also check our morgan_fingerprint_generator.py file in our repository.
morgan_fp_874	Morgan Fingerprint	https://www.rdkit.org/docs/source/rdkit.Chem.AllChem.html, also check our morgan_fingerprint_generator.py file in our repository.

Table S3. Classification results for each ML model.

Model	True Positive	False Positive	True Negative	False Negative
**Empirical rule**	324	155	35	34
**RFC**	268	56	134	90
**SVMP**	358	187	3	0
**SVMR**	327	134	56	31
**SVMS**	358	187	3	0
**XGBoost**	253	55	135	105
**Naïve-Bayes**	299	134	56	59
**ANN**	258	71	119	100
**Ensemble (RFC + XGBoost)**	293	65	125	65

Table S4. Data used to estimate the MAD of a selected set of drugs, in which GSESolver was tested.

Id (ref)	M (g/mol)	MAD (mg)	K_a (cm/s)	Surface Area (cm^2)	Solubility (mg/mL)	Residence Time (s)	Melting point (°C)	logP_N	LiProS[a]	pK_a[b]	log$D_{6.5}$	GSE log$S$	Solubility GSE (mg/mL)	MAD GSE (mg)	DMAD	GSESolver prediction
**Rizatriptan** [9]	269.34	0.9	5.10E-07	76.7	42	548	178	1.40	Use Eq. 1	9.4	-1.50	0.470546	795.879	17.061	16.161	Use GSE
**Vardenafil** [9]	488.6	0.1	5.18E-06	76.7	0.1	2517	192	1.40	Use Eq. 1	9.0	-1.10	-0.06863	417.181	417.189	417.089	Do not use GSE
**Propranolol** [9]	259.34	7.7	1.25E-05	76.7	50	161	96	3.48	Use Eq. 1	9.3	0.68	-0.88931	33.462	5.165	2.535	Use GSE
**Verapamil** [9]	454.6	4.0	8.00E-05	76.7	83	8	145	3.79	Use Eq. 2	9.6	0.20	-0.89558	57.816	2.838	1.162	Use GSE
**Nicotine** [9]	162.23	3.4	1.81E-04	76.7	1000	0.2	-79	1.17	Use Eq. 1	8.6	-0.93	2.473436	48257.772	133.842	130.442	Use GSE

Id (ref)	M (g/mol)	MAD (mg)	K_a (1/min)	Water Volume (mL)	Solubility (mg/mL)	Transit Time (min)	Melting point (°C)	logP_N	LiProS[a]	pK_a[b]	log$D_{6.5}$	GSE log$S$	Solubility GSE (mg/mL)	MAD GSE (mg)	DMAD	GSESolver prediction
**Azithromycin** [10]	749	34.02	0.001	250	0.50	270	126	4.02	Use Eq. 1	11.00	-0.48	-0.02998	699.030	47184.496	47150.476	Do not use GSE

[a] LiProS assesses the most appropriate method to calculate log D_{pH}.[11] [b] pK_a values were calculated with https://playground.calculators.cxn.io/

Table S5. Experimental, computed, and GSE-predicted data of a small set of agrochemicals, in which GSESolver was tested.[12]

Name	Type	M (g/mol)	Solubility (ppm)	logP_N	MP (°C)	S (mol/L)	logS	logS GSE	DlogS	GSESolver prediction
**Abamectin**	Chewing Pest Insecticides	1732.14	19	4.4	165.5	1.09691E-05	-4.96	-5.31	0.35	Do not use GSE
**Chlorantraniliprole**	Chewing Pest Insecticides	483.152	2.5	2.76	209	5.17436E-06	-5.29	-4.1	1.19	Do not use GSE
**Chromafenozide**	Chewing Pest Insecticides	394.513	2.2	2.7	186	5.5765E-06	-5.25	-3.81	1.44	Use GSE
**Cypermethrin**	Chewing Pest Insecticides	416.303	1.4	6.94	81	3.36294E-06	-5.47	-7	1.53	Do not use GSE
**Emamectin benzoate**	Chewing Pest Insecticides	1008.25	321	5	143.5	0.000318373	-3.5	-5.69	2.19	Do not use GSE
**Fenoxycarb**	Chewing Pest Insecticides	301.341	4	4.07	53.5	1.3274E-05	-4.88	-3.86	1.02	Use GSE
**Fipronil**	Chewing Pest Insecticides	437.151	1.8	4	203	4.11757E-06	-5.39	-5.28	0.11	Do not use GSE
**Flubendiamide**	Chewing Pest Insecticides	682.39	1	4.2	218.5	1.46544E-06	-5.83	-5.64	0.19	Do not use GSE
**Flufenoxuron**	Chewing Pest Insecticides	488.77	2	4	170.5	4.0919E-06	-5.39	-4.96	0.43	Do not use GSE
**Indoxacarb**	Chewing Pest Insecticides	527.838	1.9	4.65	88	3.59959E-06	-5.44	-4.78	0.66	Do not use GSE
**Lufenuron**	Chewing Pest Insecticides	511.152	2	5.12	168.5	3.91273E-06	-5.41	-6.06	0.65	Do not use GSE
**Metaflumizone**	Chewing Pest Insecticides	506.405	2	4.7	197	3.94941E-06	-5.4	-5.92	0.52	Do not use GSE
**Methoxyfenozide**	Chewing Pest Insecticides	368.475	4.2	3.7	207	1.13983E-05	-4.94	-5.02	0.08	Do not use GSE
**Novaluron**	Chewing Pest Insecticides	492.705	1.9	4.3	177.5	3.85626E-06	-5.41	-5.33	0.08	Use GSE
**Noviflumuron**	Chewing Pest Insecticides	529.142	2	4.94	156	3.7797E-06	-5.42	-5.75	0.33	Do not use GSE
**Spinetoram**	Chewing Pest Insecticides	748.007	2	4.1	143	2.67377E-06	-5.57	-4.78	0.79	Do not use GSE
**Spinosad**	Chewing Pest Insecticides	1477.96	4	4	91.5	2.70643E-06	-5.57	-4.17	1.4	Use GSE
**Tebufenozide**	Chewing Pest Insecticides	352.476	4.9	4.25	191	1.39017E-05	-4.86	-5.41	0.55	Do not use GSE
**Tebufenpyrad**	Chewing Pest Insecticides	333.862	2	4.93	65	5.9905E-06	-5.22	-4.83	0.39	Do not use GSE
**Teflubenzuron**	Chewing Pest Insecticides	381.112	2	4.3	219	5.2478E-06	-5.28	-5.74	0.46	Use GSE
**Tolfenpyrad**	Chewing Pest Insecticides	383.878	20	5.61	87.5	5.20999E-05	-4.28	-5.74	1.46	Do not use GSE
**Acetamiprid**	Sap-Feeding Pest Insecticides	222.678	921	0.8	99	0.004136017	-2.38	-1.04	1.34	Do not use GSE
**Clothianidin**	Sap-Feeding Pest Insecticides	249.682	223	0.7	177	0.000893136	-3.05	-1.72	1.33	Use GSE
**Cyantraniliprole**	Sap-Feeding Pest Insecticides	473.717	60	1.94	224	0.000126658	-3.9	-3.43	0.47	Do not use GSE
**Dinotefuran**	Sap-Feeding Pest Insecticides	202.213	822	-0.55	108	0.004065021	-2.39	0.22	2.61	Do not use GSE
**Flonicamid**	Sap-Feeding Pest Insecticides	229.161	1039	0.3	157	0.00453393	-2.34	-1.12	1.22	Do not use GSE

Name	Type	M (g/mol)	Solubility (ppm)	logP_N	MP (°C)	S (mol/L)	logS	logS GSE	DlogS	GSESolver prediction
**Imidacloprid**	Sap-Feeding Pest Insecticides	255.664	220	0.57	144	0.000860504	-3.07	-1.26	1.81	Use GSE
**Nitenpyram**	Sap-Feeding Pest Insecticides	270.719	1133	-0.66	82	0.004185151	-2.38	0.59	2.97	Do not use GSE
**Pymetrozine**	Sap-Feeding Pest Insecticides	217.231	215	-0.18	217	0.00098973	-3	-1.24	1.76	Use GSE
**Pyrifluquinazon**	Sap-Feeding Pest Insecticides	464.34	3	3.12	138.5	6.46078E-06	-5.19	-3.76	1.43	Use GSE
**Spirotetramat**	Sap-Feeding Pest Insecticides	373.447	41	2.51	142	0.000109788	-3.96	-3.18	0.78	Do not use GSE
**Sulfoxaflor**	Sap-Feeding Pest Insecticides	277.269	443	0.8	113	0.001597726	-2.8	-1.18	1.62	Use GSE
**Thiacloprid**	Sap-Feeding Pest Insecticides	252.728	186	0.73	136	0.000735969	-3.13	-1.34	1.79	Use GSE
**Thiamethoxam**	Sap-Feeding Pest Insecticides	291.718	240	-0.13	139	0.000822712	-3.08	-0.51	2.57	Use GSE
**Acibenzolar-S-methyl**	Fungicides	210.281	15.6	3.1	133	7.41864E-05	-4.13	-3.68	0.45	Do not use GSE
**Azoxystrobin**	Fungicides	403.393	7	2.5	116	1.73528E-05	-4.76	-2.91	1.85	Use GSE
**Benzovindiflupyr**	Fungicides	398.239	1.1	4.3	148	2.76216E-06	-5.56	-5.03	0.53	Use GSE
**Bixafen**	Fungicides	414.213	1.9	3.3	143	4.58701E-06	-5.34	-3.98	1.36	Use GSE
**Boscalid**	Fungicides	343.212	4.2	2.96	143.5	1.22373E-05	-4.91	-3.65	1.26	Use GSE
**Carbendazim**	Fungicides	191.189	5.9	1.51	304.5	3.08595E-05	-4.51	-3.81	0.7	Use GSE
**Carboxin**	Fungicides	235.306	194	2.3	91.5	0.000824458	-3.08	-2.47	0.61	Do not use GSE
**Cyproconazole**	Fungicides	291.781	124	3.1	106	0.000424976	-3.37	-3.41	0.04	Use GSE
**Cyprodinil**	Fungicides	225.294	7.5	4	76	3.32898E-05	-4.48	-4.01	0.47	Use GSE
**Epoxiconazole**	Fungicides	329.761	6.6	3.33	136.5	2.00145E-05	-4.7	-3.95	0.75	Use GSE
**Fenbuconazole**	Fungicides	336.825	5.9	3.23	126.5	1.75165E-05	-4.76	-3.75	1.01	Use GSE
**Fluopyram**	Fungicides	396.717	18.5	2.85	118	4.66327E-05	-4.33	-3.28	1.05	Use GSE
**Fluoxastrobin**	Fungicides	458.832	2.1	2.86	105.5	4.57684E-06	-5.34	-3.17	2.17	Do not use GSE
**Fluquinconazole**	Fungicides	376.178	1.9	3.24	195	5.0508E-06	-5.3	-4.44	0.86	Use GSE
**Flutolanil**	Fungicides	323.313	6.8	3.17	106	2.10323E-05	-4.68	-3.48	1.2	Use GSE
**Fluxapyroxad**	Fungicides	381.303	3.9	3.08	157	1.02281E-05	-4.99	-3.9	1.09	Use GSE
**Iprodione**	Fungicides	330.17	250	3	134	0.000757186	-3.12	-3.59	0.47	Use GSE
**Isopyrazam**	Fungicides	359.419	2.1	4.44	137	5.84276E-06	-5.23	-5.06	0.17	Use GSE
**Penflufen**	Fungicides	317.407	8.9	3.3	111	2.80397E-05	-4.55	-3.66	0.89	Use GSE

Name	Type	M (g/mol)	Solubility (ppm)	logP_N	MP (°C)	S (mol/L)	logS	logS GSE	DlogS	GSESolver prediction
**Picoxystrobin**	Fungicides	367.322	2.1	3.6	75	5.71705E-06	-5.24	-3.6	1.64	Use GSE
**Prochloraz**	Fungicides	376.67	198	3.53	48	0.000525659	-3.28	-3.26	0.02	Use GSE
**Propiconazole**	Fungicides	342.225	428	3.72	-23	0.001250639	-2.9	-2.74	0.16	Do not use GSE
**Prothioconazole**	Fungicides	344.265	68.9	3.82	141.5	0.000200137	-3.7	-4.49	0.79	Use GSE
**Sedaxane**	Fungicides	331.365	9.6	3.3	121	2.89711E-05	-4.54	-3.76	0.78	Use GSE
**Tebuconazole**	Fungicides	307.824	39	3.7	105	0.000126696	-3.9	-4	0.1	Use GSE
**Thiophanate-methyl**	Fungicides	342.399	28.3	1.44	165	8.26521E-05	-4.08	-2.34	1.74	Use GSE
**Trifloxystrobin**	Fungicides	408.375	1.8	4.5	73	4.40771E-06	-5.36	-4.48	0.88	Use GSE
**2,4-D**	Post-Emergency Herbicides	221.039	669	0.177	140	0.003026615	-2.52	-0.83	1.69	Use GSE
**Aminopyralid**	Post-Emergency Herbicides	207.016	977	-2.87	164	0.004719442	-2.33	1.98	4.31	Use GSE
**Bispyribac-sodium**	Post-Emergency Herbicides	452.354	104	-1.03	223.5	0.000229908	-3.64	-0.46	3.18	Use GSE
**Clopyralid**	Post-Emergency Herbicides	192.001	852	-2.63	331.5	0.004437477	-2.35	0.06	2.41	Use GSE
**Dicamba**	Post-Emergency Herbicides	221.039	1034	-1.88	115	0.004677908	-2.33	1.48	3.81	Use GSE
**Fluroxypyr**	Post-Emergency Herbicides	255.032	630	-1.24	232.5	0.002470278	-2.61	-0.34	2.27	Use GSE
**Fluroxypyr-meptyl**	Post-Emergency Herbicides	367.247	1	5.04	59	2.72296E-06	-5.56	-4.88	0.68	Use GSE
**Halauxifen**	Post-Emergency Herbicides	331.129	127	-0.83	168	0.000383536	-3.42	-0.1	3.32	Use GSE
**Halauxifen-methyl**	Post-Emergency Herbicides	345.156	3	3.76	146	8.69172E-06	-5.06	-4.47	0.59	Use GSE
**Imazamox**	Post-Emergency Herbicides	305.333	366	0.73	166	0.001198691	-2.92	-1.64	1.28	Use GSE
**Mesosulfuron-methyl**	Post-Emergency Herbicides	503.512	55	-0.48	195	0.000109233	-3.96	-0.72	3.24	Use GSE
**Mesotrione**	Post-Emergency Herbicides	339.323	212	-1	165	0.000624773	-3.2	0.1	3.3	Use GSE
**Pinoxaden**	Post-Emergency Herbicides	400.517	201	3.2	121	0.000501851	-3.3	-3.66	0.36	Use GSE
**Pyrasulfotole**	Post-Emergency Herbicides	362.327	339	-1.36	201	0.000935619	-3.03	0.1	3.13	Do not use GSE
**Pyroxsulam**	Post-Emergency Herbicides	434.354	155	-1.01	208	0.000356852	-3.45	-0.32	3.13	Use GSE
**Quinclorac**	Post-Emergency Herbicides	242.061	256	-0.74	274	0.001057585	-2.98	-1.25	1.73	Use GSE

Name	Type	M (g/mol)	Solubility (ppm)	logP_N	MP (°C)	S (mol/L)	logS	logS GSE	DlogS	GSESolver prediction
**Thiencarbazone-methyl**	Post-Emergency Herbicides	390.396	245	-1.98	206	0.000627568	-3.2	0.67	3.87	Use GSE
**Tralkoxydim**	Post-Emergency Herbicides	329.438	140	2.1	106	0.000424966	-3.37	-2.41	0.96	Do not use GSE
**Tribenuron-methyl**	Post-Emergency Herbicides	395.395	211	0.78	142	0.000533644	-3.27	-1.45	1.82	Use GSE
**Atrazine**	Post-Emergency Herbicides	215.687	32	2.5	176	0.000148363	-3.83	-3.51	0.32	Use GSE
**Bentazone**	Post-Emergency Herbicides	240.282	792	-0.46	138	0.003296127	-2.48	-0.17	2.31	Use GSE
**Bromoxynil**	Post-Emergency Herbicides	276.915	152	1.04	194.5	0.000548905	-3.26	-2.24	1.02	Do not use GSE
**Carfentrazone-ethyl**	Post-Emergency Herbicides	412.194	28	3.36	-22	6.79292E-05	-4.17	-2.39	1.78	Use GSE
**Diflufenican**	Post-Emergency Herbicides	394.298	2	4.2	159	5.07231E-06	-5.29	-5.04	0.25	Use GSE
**Metribuzin**	Post-Emergency Herbicides	214.292	277	1.6	126	0.001292629	-2.89	-2.11	0.78	Use GSE

Technical information about the trained Machine Learning Models

t-SNE hyperparameters:

dims = 2, perplexity = 25, verbose = TRUE, max_iter = 1500, check_duplicates = FALSE

Random Forest Classifier's hyperparameter tuning

- **'n_estimators'** (number of trees): 100
- **'min_samples_split'** (sample minimum to create a node split): 5
- **'min_samples_leaf'**: 2
- **'max_depth'**: 30
- **'criterion'** (node division criterion): 'entropy' [6]

Support Vector Machine's hyperparameter tuning

Polynomial kernel (SVMP):

- **'gamma'** (decision boundary): 'scale' $\left(\frac{1}{n_{descriptors} \cdot s_X}\right)$
- **'degree'** (polynomial degree): 4
- **'coef0'** (*y* axis intersection): 0.0
- **'C'** (regularization parameter): 0.1

Radial kernel (SVMR):

- **'gamma'**: 'auto' $\left(\frac{1}{n_{descriptors}}\right)$
- **'C'**: 10

Sigmoid kernel (SVMS):

- **'gamma'**: 'scale' $\left(\frac{1}{n_{descriptors} \cdot s_X}\right)$
- **'coef0'**: 0.1
- **'C'**: 1

XGBoost's hyperparameter tuning[7]

- **'subsample'** (subsample used to 'grow trees'): 0.6
- **'reg_lambda'** (weighing regulator for each leaf): 0
- **'reg_alpha'** (weighing regulator for each leaf):: 0.05,
- **'n_estimators'** (number of trees): 300,
- **'min_child_weight'** (minimum weight to execute a node split): 3,
- **'max_depth'** (maximum depth of a tree): 9,
- **'learning_rate'**: 0.01,
- **'gamma'** (error reduction between splits): 0.3,
- **'colsample_bytree'** (fraction of descriptors per tree): 0.7

Naïve-Bayes' model architecture

GaussianNB's algorithm uses Bayes' theorem to calculate the probability of an event A, given event B:[8]

$$P(A|B) = \frac{P(B|A)P(A)}{P(B)}$$

This algorithm computes the probability of the dependent variable for each descriptor, assuming all variables are completely independent (hence the term *naïve*).

$$P(y|x_1, \dots, x_n) = \frac{P(y)\prod_{i=1}^{n} P(x_1, \dots, x_n|y)}{P(x_1, \dots, x_n)}$$

GaussianNB calculates probability from each descriptor, given the dependent variable *y* assuming a normal distribution (hence the term *Gaussian*).

$$P(x_i|y) = \frac{1}{\sqrt{2\pi\sigma_y^2}} e^{-\frac{(x_i-\mu_y)^2}{2\sigma_y^2}}$$

The probability of *y*, given the selected descriptors is transformed to an output value $\hat{y}$ by:

$$\hat{y} = \underset{y}{\operatorname{argmax}} P(y|x_1, \dots, x_n)$$

Artificial Neural Network

All continuous input features were standardised using StandardScaler (zero mean, unit variance) prior to model training. The final artificial neural network (ANN) architecture was as follows:

- **Input layer**: Input(shape = n_descs) – one node per descriptor.
- **Hidden layer 1**: Dense(32, activation = 'relu', kernel_regularizer = l2(1e-3)) – 32 nodes with ReLU activation and L2 regularisation (penalty factor 1×10^{-3}) to mitigate overfitting.
- **BatchNormalization()** – normalised the activations to accelerate and stabilise training.
- **Dropout(0.5)** – randomly deactivated 50 % of neurons during training.
- **Hidden layer 2**: Dense(16, activation = 'relu', kernel_regularizer = l2(1e-3)) – 16 nodes with ReLU activation and L2 regularisation.
- **BatchNormalization()** – as above.
- **Dropout(0.4)** – randomly deactivated 40 % of neurons.
- **Output layer**: Dense(1, activation = 'sigmoid') – single node with sigmoid activation, appropriate for binary classification.

To address the inherent class imbalance, we employed a **focal loss** function (with $\gamma = 2.0$ and $\alpha = 0.75$) instead of class weighting. This loss downweighs the contribution of easy-to-classify examples, forcing the model to focus on hard, misclassified instances, and has been shown to be effective for imbalanced

datasets. The standard class weights were set to 1:1 (i.e., no additional weighting) in the class_weight argument, as the focal loss itself handled the imbalance.

The model was compiled with the **Adam** optimizer (learning rate = 5×10^{-4}), using focal loss as the objective, and tracked both **accuracy** and the **area under the ROC curve (AUC)** as performance metrics (on the validation set).

Training proceeded for a maximum of **1000 epochs** with a **batch size of 32**. Two callbacks were employed:

- **EarlyStopping** – monitored val_auc with a patience of 30 epochs, restoring the best weights to avoid overfitting.
- **ReduceLROnPlateau** – reduced the learning rate by a factor of 0.5 if val_auc did not improve for 15 consecutive epochs, with a minimum learning rate of 1×10^{-7}.

The model was wrapped using KerasClassifier from the scikeras package to facilitate cross-validation.

References:

1. Kier, L. B. Indexes of molecular shape from chemical graphs. *Med. Res. Rev.* **7**, 417–440 (1987).
2. Wildman, S. A. & Crippen, G. M. Prediction of Physicochemical Parameters by Atomic Contributions. *J. Chem. Inf. Comput. Sci.* **39**, 868–873 (1999).
3. Hall, L. H. & Kier, L. B. Electrotopological State Indices for Atom Types: A Novel Combination of Electronic, Topological, and Valence State Information. *J. Chem. Inf. Comput. Sci.* **35**, 1039–1045 (1995).
4. Gasteiger, J. & Marsili, M. Iterative partial equalization of orbital electronegativity—a rapid access to atomic charges. *Tetrahedron* **36**, 3219–3228 (1980).
5. Nguyen, K. T., Blum, L. C., van Deursen, R. & Reymond, J.-L. Classification of Organic Molecules by Molecular Quantum Numbers. *ChemMedChem* **4**, 1803–1805 (2009).
6. Shannon, C. E. A Mathematical Theory of Communication. *Bell System Technical Journal* **27**, 379–423 (1948).
7. XGBoost Parameters — xgboost 3.1.1 documentation. https://xgboost.readthedocs.io/en/stable/parameter.html.
8. scikit-learn: machine learning in Python — scikit-learn 1.7.2 documentation. https://scikit-learn.org/stable/.
9. Yang, Z. *et al.* Application of *in vitro* transmucosal permeability, dose number, and maximum absorbable dose for biopharmaceutics assessment during early drug development for intraoral delivery. *International Journal of Pharmaceutics* **503**, 78–89 (2016).
10. Curatolo, W. J. Physical chemical properties of oral drug candidates in the discovery and exploratory development settings. *Pharmaceutical Science & Technology Today* **1**, 387–393 (1998).
11. Bertsch-Aguilar, E. *et al.* LiProS: Findable, Accessible, Interoperable, and Reusable Data Simulation Workflow to Predict Accurate Lipophilicity Profiles for Small Molecules. *Molecular Informatics* **44**, e202500136 (2025).
12. Zhang, Y. *et al.* Physicochemical property guidelines for modern agrochemicals. *Pest Management Science* **74**, 1979–1991 (2018).
